# The Rise of Chalcohalide Solar Cells: Comprehensive Insights From Materials to Devices

**DOI:** 10.1002/advs.202413131

**Published:** 2025-04-17

**Authors:** Hongrui Zhang, Yiming Xia, Yangfan Zhang, Uma V. Ghorpade, Mingrui He, Seung Wook Shin, Xiaojing Hao, Mahesh P. Suryawanshi

**Affiliations:** ^1^ School of Photovoltaic and Renewable Energy Engineering University of New South Wales Sydney New South Wales 2052 Australia; ^2^ School of Chemical Engineering University of New South Wales Sydney New South Wales 2052 Australia; ^3^ Future Agricultural Research Division Rural Research Institute Korea Rural Community Corporation Ansan‐si 15634 Republic of Korea

**Keywords:** metal chalcohalides, perovskite‐inspired semiconductors, solar absorber, thin film photovoltaics

## Abstract

While lead‐halide perovskites achieve high efficiencies, their toxicity and instability drive the search for safer materials. Chalcohalides, combining chalcogen and halogen anions in versatile structures, emerge as earth‐abundant, nontoxic alternatives for efficient photovoltaic (PV) devices. A wide variety of chalcohalide materials, including pnictogen metals‐, post‐transition metals‐, mixed‐metals‐ and organic–inorganic metals‐based chalcohalides, offer diverse structural, compositional, and optoelectronic characteristics. Some of these materials have already been experimentally synthesized and integrated into PV devices, achieving efficiencies of 4–6%, while others remain theoretically predicated. Despite these advancements, significant challenges must be addressed to fully realize the potential of chalcohalides as next‐generation PV absorbers. This review provides a comprehensive insight of the fundamental properties of chalcohalide materials, emphasizing their unique structures, highly interesting optoelectronic and dielectric properties, to fuel further research and guide the development of high‐efficiency chalcohalide solar cells. Various synthesis techniques are discussed, highlighting important and potentially overlooked strategies for fabricating complex quaternary and pentanary chalcohalide materials. Additionally, the working principles of different device structures and recent advances in fabricating efficient chalcohalide solar cells are covered. We hope that this review inspires further exciting research, innovative approaches, and breakthroughs in the field of chalcohalide materials.

## Graphical abstract

This review provides a detailed overview of recent advancements in chalcohalide solar cells, focusing on structure design, material chemistry, synthesis methods, and device optimization. It also highlights future directions for improving device efficiency through innovative device architectures and synthesis techniques, addressing key challenges in sustainable energy production.

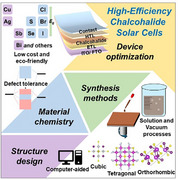

## 1 Introduction

Hybrid organic–inorganic lead halide perovskite materials have revolutionized photovoltaics (PV) with exceptional properties like high absorption coefficients, long charge carrier diffusion lengths, and minimal deep defects.^[^ ^1^ ^]^ These advancements have led to remarkable single‐junction solar cell efficiencies of up to 26%^[^ ^2^ ^]^ and the promising development of perovskite‐silicon tandem devices, which have achieved efficiencies exceeding 30%,^[^ ^3^ ^]^ surpassing the limits of silicon technology alone. Additionally, the low‐temperature deposition methods for perovskites facilitate their integration with existing silicon subcells.^[^ ^4^ ^]^ However, the long‐term stability of halide perovskite solar cells remains a significant challenge, as they are prone to degradation under various stressors.^[^ ^5^ ^]^ Ensuring the longevity of these cells to match or exceed the durability of current silicon technologies is crucial for their commercial viability. Enhancing the stability of halide perovskites through encapsulation is possible, but modifying their intrinsic properties is essential for a lasting solution. The toxicity of lead in these halide perovskites also hinders their commercial viability,^[^ ^6^ ^]^ prompting current research to explore improvements without losing their advantageous characteristics. To ensure long‐term sustainability and reduce the risks associated with depending too heavily on perovskite technology, it is crucial to promote collaborative research and diversify investments across multiple PV materials.

Chalcogenides, another promising family of PV materials, have already demonstrated high performance in single junction solar cells using Cu(In, Ga)(S, Se)_2_ (CIGS) and CdTe absorbers.^[^ ^3^, ^7^ ^]^ However, the scarcity and toxicity issues associated with In, Te, and Cd have led to the development of Cu_2_ZnSn(S,Se)_4_ (CZTSSe), an earth‐abundant, nontoxic, and inexpensive alternate absorber materials with efficiency reaching ≈15%.^[^ ^7^, ^8^ ^]^ Despite its promise, high deep‐level defects, some lattice instabilities and secondary phase formation during synthesis in CZTSSe remain challenges.^[^ ^8^, ^9^ ^]^ In the search for defect‐tolerant absorbers with high stability and to avoid toxic Pb, chalcogenides adopting perovskite structures (referred to as “chalcogenide perovskites”) have gained significant attention in the PV community.^[^ ^10^ ^]^ These chalcogenide perovskites exhibit exceptional optoelectronic and chemical properties, including outstanding stability and intense photoluminescence, prerequisites for high‐efficiency PV devices.^[^ ^11^ ^]^ However, their synthesis chemistry differs from halide counterparts, leading to challenges in thin film formation at low temperatures.^[^ ^12^ ^]^ While these chalcogenide absorbers offer superior environmental stability compared to halide perovskites, this advantage often necessitates higher synthesis temperatures (>450 °C for chalcogenide perovskites). None of these absorbers have shown primary indications of high PV potential, such as a high photoluminescence quantum yield, further highlighting the need for alternative light‐absorbing materials with excellent optoelectronic and defect‐tolerant properties like halide perovskites and exceptional stability like chalcogenide. This remains an incredibly timely and vibrant area of research, capturing the attention and enthusiasm of the PV community like never before.

Chalcohalide materials are an emerging class of inorganic semiconductors, also known as “multi‐anion or mixed anion” semiconductors, often overlooked for their potential in PV devices.^[^ ^13^ ^]^ Defined as MChX, where M is one or more metal cations (e.g., Bi, Sb, Ag, Cu, Pb, and Sn) and Ch and X are one or more chalcogen (e.g., S, Se, and Te) and halogen (e.g., I, Br, Cl) anions, respectively. The presence of both divalent chalcogen and monovalent halogen anions in these chalcohalides offers tunability over structural and optoelectronic properties as well as phase stability.^[^ ^14^ ^]^ Importantly, trivalent metal cations in these chalcohalides with a 5s^2^ electronic configuration similar to Pb^2+^ produce PV materials with a defect‐tolerant electronic structure similar to Pb‐halide perovskite materials.^[^ ^14^, ^15^ ^]^ Additionally, their peculiar electronic structures, with a highly dispersive character of the valence band (VB) and conduction band (CB), provide high charge carrier mobilities and defect tolerance. These materials are more thermodynamically stable,^[^ ^16^ ^]^ allowing low‐temperature synthesis methods, just like halide perovskites, for materials design by avoiding halogen volatility, further favoring their practical applications. These outstanding properties have prompted investigations into their fundamental structure and optoelectronic properties and integration into different devices such as room‐temperature radiation detectors,^[^ ^17^ ^]^ photodetectors,^[^ ^18^ ^]^ light‐emitting diodes (LEDs),^[^ ^19^ ^]^ supercapacitors,^[^ ^20^ ^]^ batteries,^[^ ^21^ ^]^ photocatalysis,^[^ ^13^, ^22^ ^]^ and solar cells.^[^ ^14^, ^16^, ^23^ ^]^ Few reviews have highlighted the promise of chalcohalide materials for energy devices, briefly describing their device applications.^[^ ^13^, ^23^, ^24^ ^]^ For example, Ghorpade et al.^[^ ^13a^ ^]^ provide an overview of the design, synthesis, and applications of chalcohalide materials as emerging semiconductors for energy conversion and storage devices. Another review by Shyamal and Pradhan focuses on the applications of chalcohalide materials in photocatalysis including water splitting, pollutant removal, and CO₂ reduction.^[^ ^13b^ ^]^ In 2020, Choi et al.^[^ ^24a,b^ ^]^ comprehensively reviewed Sb/Bi chalcohalide solar cells, covering their physical properties and device performance, followed by recent developments on Sb/Bi chalcohalides and Bi oxyhalides. Recently, Giulia and coworkers reviewed recent progress in quaternary chalcohalides, classifying their structures into AMChX₂, A₂MCh₂X₃, AMCh₃X, and other AMChX materials, providing insights into the synthesis concept of quaternary chalcohalides.^[^ ^23f^ ^]^ These published reviews have made important contributions to this field. However, a growing interest in these materials specifically for PV applications has inspired us to put forward a more extensive review focused on their basic fundamentals including structure, optoelectronic, electrical, and dielectric as well as defect properties as well as PV device working principles and recent breakthroughs in the PV field. Finally, we also explore the possible uses of chalcohalides as top‐ and bottom‐cells for tandem PV, an aspect not often discussed in previous reviews.

This review aims to kick‐start a serious investigation of chalcohalide materials for PV applications by clarifying some of the early misunderstandings of fundamentals, mainly defect properties from a PV device point of view, and providing guidance for scientists looking to contribute to this interesting class of semiconductor materials. Following the introduction (Section 1), we briefly discuss the historical background of chalcohalide materials, covering early work on their synthesis and properties to their current state‐of‐the‐art from a PV devices perspective (Section 2). Next, we highlight their fundamental attributes, including structural chemistry, optoelectronic, dielectric, and defect tolerance properties prerequisites for high‐efficiency PV devices (Section 3). We also discuss different PV device structures employed and their working principles, which are important to understanding chalcohalide solar cell development. We comprehensively discuss the various synthesis methods adopted for chalcohalide materials and further highlight major achievements in their PV device applications. Finally, we also explore the possible uses of chalcohalides as top‐ and bottom‐cells for tandem PV, an aspect not often discussed in previous reviews. Beyond summarizing synthesis developments and device performances, we provide challenges and key directions for preparing high‐quality chalcohalide materials and fabricating high‐efficiency PV devices.

## 2 Historical Background of Chalcohalides

The most widely studied chalcohalide materials are SbSI and BiSI, which are also receiving great attention for solar cells recently. Although the synthesis of SbSI dates back to the 18th century, its rhombic–bipyramidal structure was first revealed by E. Donges in 1950.^[^ ^25^ ^]^ This material is the first material discovered to be ferroelectric and photoconductive in the family of chalcohalide materials. The photoelectric properties were first reported by Nitsche and Mertz in 1960,^[^ ^26^ ^]^ whereas ferroelectricity was first revealed by Fatuzzo et al.^[^ ^27^ ^]^ in 1962. The first synthesis of orthorhombic BiSI material was reported by Francois and Delwaulle in 1936,^[^ ^28^ ^]^ however the property‐based analysis remained unexplored until 1950. Interestingly, the first photoelectric response of this material was quantified in 1960, revealing the maximum photo response at 785 nm.^[^ ^26^ ^]^ The measurement of the spectral distribution of internal photoconductivity of this material further confirmed the anisotropy in the PV effect. The ferroelectricity and its transition in Sb‐based chalcohalides, which results in one phase with spontaneous polarization. This indicates that these materials can be an ideal candidate for exhibiting bulk and poly‐crystalline ferroelectric effects, which has piqued the interest of researchers to further explore this family of materials.

**Figure 1:**
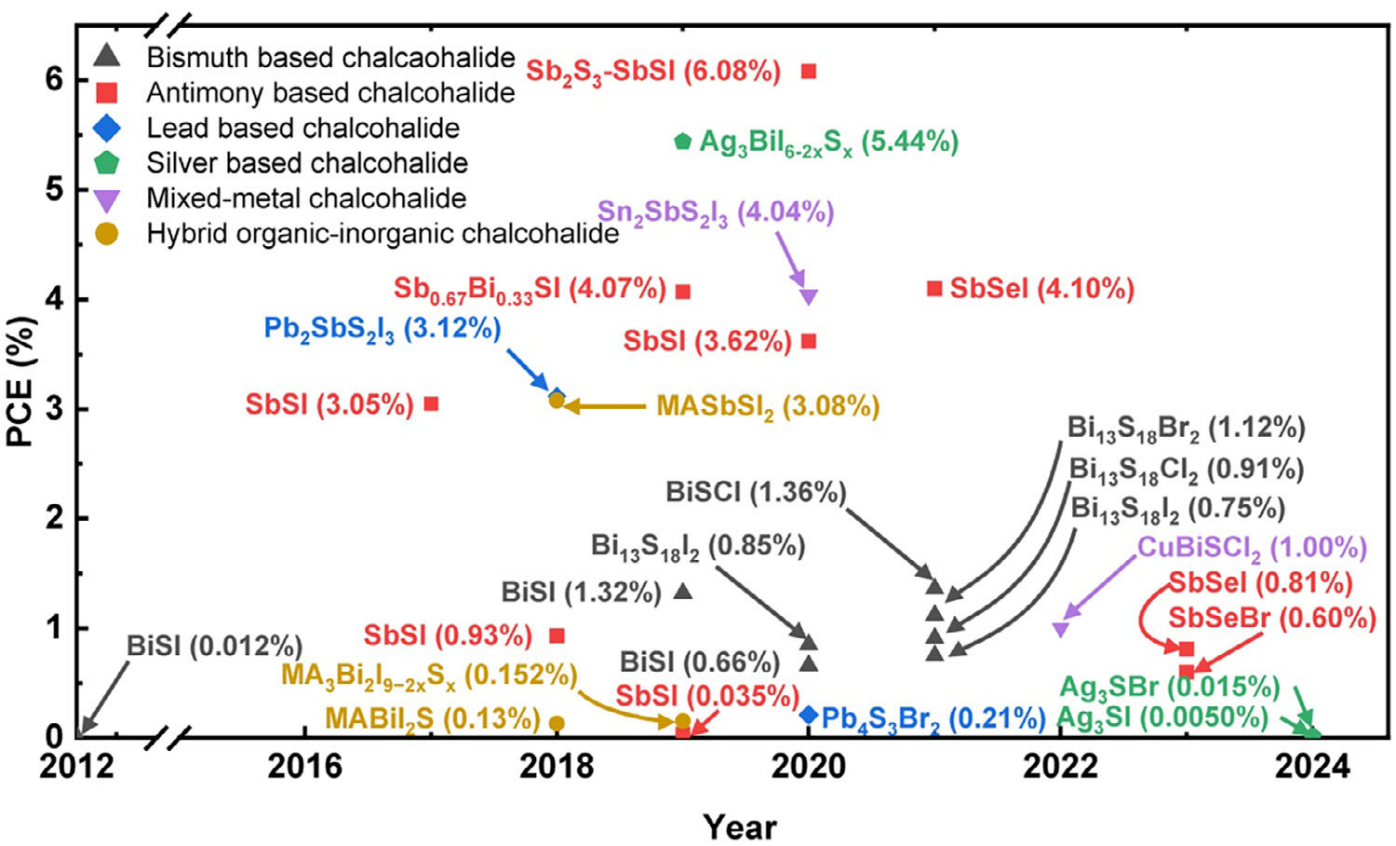
The evolution of device efficiencies of various chalcohalide solar cells.

Despite such outstanding properties, chalcohalide materials have been mostly overlooked as promising candidates for PV application until recently. The first working chalcohalide photoelectrochemical (PEC) cell was reported in 2012 by Mullins and colleagues showing photocurrent density up to 5 mA cm^−2^ with an open‐circuit voltage V_oc_ of 0.37 V.^[^ ^23b^ ^]^ Inspired by these initial promising device results, the first working solid‐state heterojunction device based on n‐type BiSI absorber and p‐type CuSCN window layer was demonstrated in the same year, however, the device had a very low efficiency of ≈0.01% due to charge carrier recombination in BiSI and light scattering by the CuSCN layer.^[^ ^23a^ ^]^ In addition, BiSI absorber had a mean hole diffusion length of ≈5 × 10^−6^ cm (≈50 nm), which is significantly lower than the most common solar cell materials,^[^ ^29^ ^]^ which is mostly likely the reason why Bi‐based chalcohalide materials have received less attention as solar absorber materials. On the other hand, 6 years later Seok's group at UNIST, South Korea, reported a 3.05% efficient SbSI solar cell that retained more than 93% of its original power conversion efficiency (PCE) after 15 days without encapsulation when stored in different humidity environments.^[^ ^23d^ ^]^ This efficiency was further improved to 4.07% by controlling the bandgap and transport properties of SbSI absorber via partially substituting Sb with Bi.^[^ ^30^ ^]^ This initial promising efficiency and stability of Sb‐based chalcohalide absorbers further triggered research on chalcohalide solar cells with several reports on device efficiencies for quaternary chalcohalide solar cells based on Pb_2_SbS_2_I_3_, Sn_2_SbS_2_I_3_, Ag_3_BiI_6‐2x_S_x_, and CuBiSCl_2_ have been reported.^[^ ^23^, ^31^ ^]^ The evolution of solar cell efficiencies of all these chalcohalide materials is presented in **Figure** **1**.

## 3 Basic Fundamentals of Chalcohalides

Chalcohalides emerge as promising candidates as absorbers for solar cells, owing to their distinct optoelectronic attributes.^[^ ^13^, ^23^, ^24^, ^32^ ^]^ Despite their demonstrated potential, it is still crucial to comprehensively understand their fundamental characteristics to fully utilize their potential.

Unlike other PV technologies, chalcohalide materials exhibit diverse compositions, making systematic classification crucial for material discovery and screening. Based on metal cation type and composition, chalcohalides can be broadly categorized into four groups: 1) heavy pnictogen chalcohalides (Bi and Sb), 2) transition and post‐transition metal chalcohalides (Cu, Sn, Pb, Ag, Na and others), 3) mixed‐metal chalcohalides (combinations of Bi or Sb with other metal cations), and 4) hybrid organic–inorganic chalcohalides (which incorporate organic cations into the chalcohalide framework).

Beyond these known categories, many chalcohalide compounds remain undiscovered. To accelerate their discovery, machine learning and deep learning can be employed for initial screening, followed by using density functional theory (DFT) calculations for preliminary validation.^[^ ^13a^ ^]^ In this section, we analyze the key properties of chalcohalides, including bandgap, electrical and dielectric characteristics, crystal structures, and defect states. **Table** **1** summarizes the fundamental properties of representative chalcohalide materials. By integrating theoretical and experimental insights, we explore the structure–property–performance relationships in these materials and their implications for solar cell applications.

### 3.1 Crystal Structure and Phase Transition

#### 3.1.1 Heavy Pnictogen Chalcohalides

**Figure 2:**
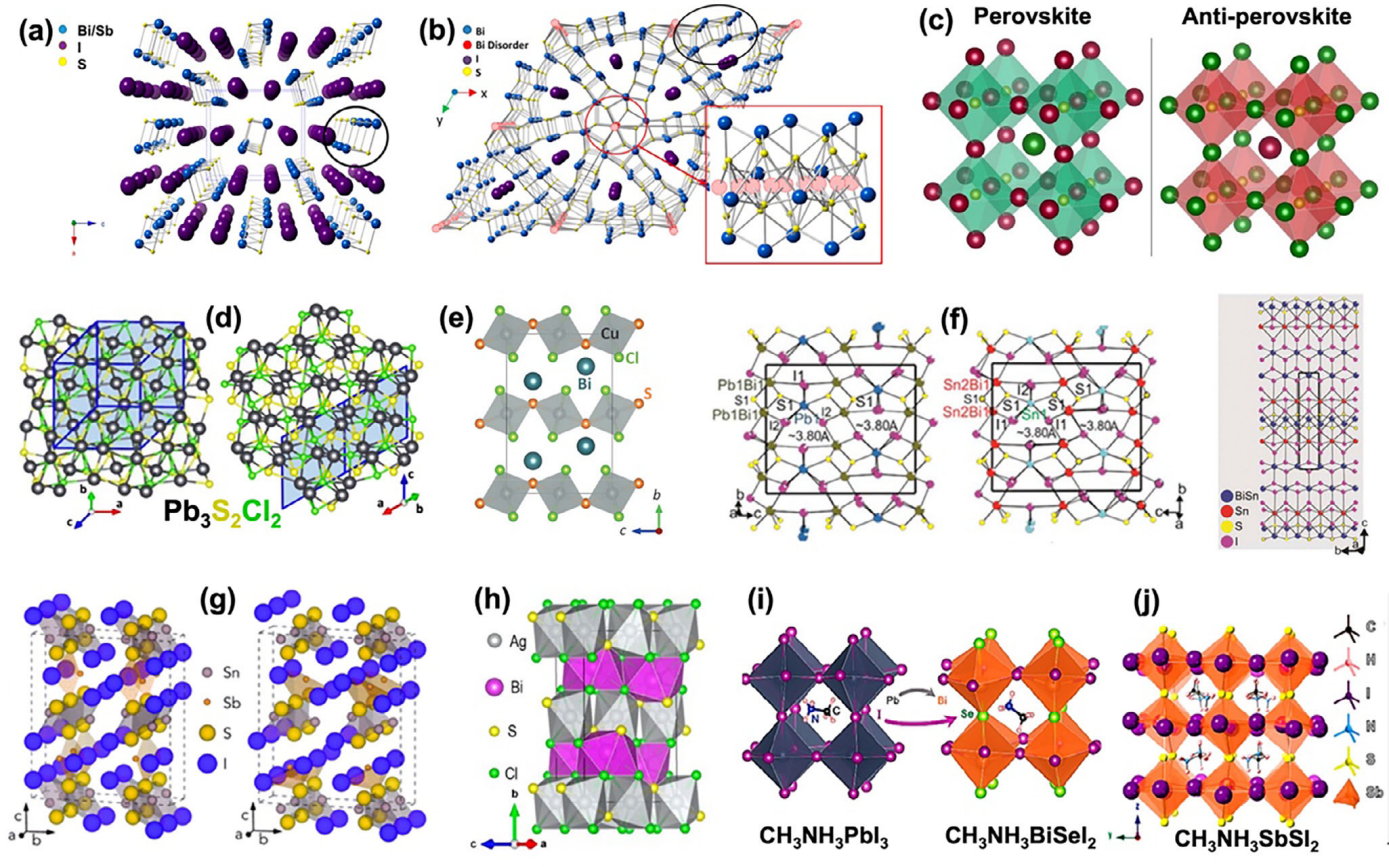
a) Structure of BiSI looking down the *b*‐axis at the *ac*‐plane. The black circle highlights the Bi–S 1‐D chain.^[^ ^35a^ ^]^ b) Averaged cell representation (ACR) of the hexagonal *P*6_3_ structure of Bi_13_S_18_I_2_ viewed down the *c*‐axis. Inset: normal to the *c*‐axis to highlight bismuth (disordered/averaged dimer) sites, in hatched red.^[^ ^36a^ ^]^ c) Schematic representation of the perovskite and antiperovskite crystal structure. Both are isostructural but with anions in place of cations and vice versa.^[^ ^24c^ ^]^ d) Two views of the Cc monoclinic Pb_3_S_2_Cl_2_ structure ([100] left, [111] right).^[^ ^23i^ ^]^ e) Atomic structure of CuBiSCl_2_.^[^ ^23h^ ^]^ f) Crystal structures of Pb_2_BiS_2_I_3_ (left) and Sn_2_BiSI_5_ (middle, right).^[^ ^43^ ^]^ g) Calculated crystal structures for Cmcm and Cmc2_1_ polymorphs of Sn_2_SbS_2_I_3_.^[^ ^16c^ ^]^ h) Crystal structure of AgBiSCl_2_, highlighting the coordination of both Ag and Bi atoms and the formation of alternate layers.^[^ ^47a^ ^]^ i) Atomic structures of CH_3_NH_3_PbI_3_ (left) and CH_3_NH_3_BiSeI_2_ (right).^[^ ^58^ ^]^ j) Crystal structure of CH_3_NH_3_SbSI_2_.^[^ ^59^ ^]^ Reproduced with permission.^[^ ^35a^ ^]^ Copyright 2017, American Chemical Society. Reproduced with permission.^[^ ^36a^ ^]^ Copyright 2017, American Chemical Society. Reproduced with permission.^[^ ^24c^ ^]^ Copyright 2022, Wiley. Reproduced with permission.^[^ ^23i^ ^]^ Copyright 2020, Nature. Reproduced with permission.^[^ ^23h^ ^]^ Copyright 2022, Wiley. Reproduced with permission.^[^ ^43^ ^]^ Copyright 2016, American Chemical Society. Reproduced with permission.^[^ ^16c^ ^]^ Copyright 2021, Royal Society of Chemistry. Reproduced with permission.^[^ ^47a^ ^]^ Copyright 2023, American Chemical Society. Reproduced with permission.^[^ ^58^ ^]^ Copyright 2016, Royal Society of Chemistry. Reproduced with permission.^[^ ^59^ ^]^ Copyright 2018, American Chemical Society.

For ternary chalcohalide compositions, the absorbers of heavy pnictogen solar cells can be represented by the formula M^III^ChX, where M^III^ = Sb or Bi, Ch = S, Se, or Te and X = Cl, Br, or I. Reflecting their periodic table groupings, these materials are commonly categorized as group V−VI−VII materials.^[^ ^23f^ ^]^ The exclusion of arsenic (As) from the heavy pnictogen elements composition is attributed to its significant toxicity and adverse environmental impact.^[^ ^33^ ^]^ Among these materials, most of the Bi‐ and Sb‐based materials (BiSX, BiSeX, SbSX, SbSeX) exhibit needle‐shaped pseudo‐1D structures with an orthodromic crystal system (Pnma or Pnam space group).^[^ ^24^, ^34^ ^]^ In materials such as SbSI and BiSI, a single chalcogen atom is substituted by two halogen atoms, resulting in the division of the M_4_S_6_ chains, typical in Sb_2_S_3_ or Bi_2_S_3_, into two separate [M_2_S_2_I_2_]_n_ chains growing parallel along the *c*‐axis, with parallel chains joined together by van der Waals forces.^[^ ^13^, ^24^ ^]^ This structural configuration, as shown in **Figure** **2**a, presents challenges in fabricating solar cell devices using these materials into planar devices.^[^ ^24^, ^35^ ^]^ In detail, SbSI undergoes a ferroelectric phase transition from Pnma to Pna2₁ ≈295 K, which influences the stability of crystal structure. In the mixed Bi_1‐x_Sb_x_SI system, phase segregation occurs during synthesis, leading to spatial variations in composition across different crystals. This inhomogeneity can alter charge transport and carrier dynamics due to local variations in optoelectronic properties. Moreover, the inherent 1D nanorod structure presents challenges for efficient charrier extraction in solar cells. Anisotropic charge transport, where carrier mobility varies along different crystallographic orientations, can further limit device performance. Considering the challenges posed by the needle‐shaped pseudo‐1D structures, it may be beneficial to investigate novel substrate designs or deposition techniques that could better align with these structures, thereby improving material integration and overall device performance. Abe et al. first reported the transition from oxyhalides to chalcohalides, achieved by introducing H_2_S into BiOI at low temperatures. This transformation is facilitated by the intercalation of H₂S molecules into the interlayer spaces of the Sillén‐phase BiOX structure. In this process, H₂S reacts with O^2^⁻ in the [Bi₂O₂]^2^⁺ layers, enabling a smooth structural transformation.^[^ ^15b^ ^]^

**Table 1:** Representative chalcohalide materials properties.

Type	Material	Crystal structure	Space group	Bandgap (Exp.) [eV]	Bandgap (Theory) [eV]	Band position nature	Conductivity type	Effective mass [me*,mh*]
Heavy Pnictogen Metal Chalcohalides	BiSX (X = Br, I, Cl)^[^ ^23a^ ^]^	orthorhombic	Pnma	1.57–1.95	1.6–1.87	direct/indirect	n‐type	e:2.91–6.21 h:0.24–0.53
	BiSeX (X = Br, I) ^[^ ^13^, ^37^ ^]^	orthorhombic	Pnma	1.3–1.54	1.03–1.45	direct/indirect	n‐type, p‐type	e:0.53–0.57 h:2.19–2.98
	BiS_1‐x_Se_x_I^[^ ^23a^ ^]^	orthorhombic	Pnma	1.48–1.63 (X = 0‐0.4)	–	direct	n‐type	–
	Bi_13_S_18_X_2_^[^ ^38^ ^]^	hexagonal	P3	0.76–1.34	0.6−0.91(Bi_13_S_18_I_2_)	direct/indirect	n‐type	–
	SbSX (I, Br)^[^ ^23^, ^39^ ^]^	orthorhombic	Pnma	2.11–2.31	2.15 (SbSBr)	indirect	p‐type	e:0.43–0.51 h:0.57–0.64
	SbSeX (X = I, Br)^[^ ^40^ ^]^	orthorhombic	Pnma	1.74(SbSeI)	1.29−1.67	direct/indirect	n‐type	e:0.54 h:0.58(SbSeI)
	BiTeX (X = Cl, Br, I)^[^ ^34^ ^]^	Hexagonal (Cl) trigonal (Br) trigonal (I)	P6_3_mc P3m1 P3m1	0.33–0.72	0.28–1.38	direct/indirect	n‐type	–
Transition and Post‐transition Metal Chalcohalides	Pb_4_S_3_I_2_^[^ ^41^ ^]^	orthorhombic	Pnma	1.5	1.84/1.65	direct/indirect	n‐type	–
	Pb_4_S_3_Br_2_ Pb_3_S_2_Cl_2_^[^ ^23^ ^i]^	orthorhombic orthorhombic	Pnma Pnma	1.91 1.76	1.98	indirect		
	Ag_3_SBr Ag_3_SI^[^ ^23j^ ^]^	cubic(anti‐perovskite) cubic(anti‐perovskite)	Pm3_m Pm3_m	0.9–1.0	–	indirect	–	–
	Cd_4_SF_6_ Cd_5_S_4_Cl_2_ Sn_5_S_4_Cl_2_ Sn_4_SF_6_^[^ ^42^ ^]^	tetragonal monoclinic orthorhombic, triclinic trigonal, orthorhombic, triclinic	R3_m Cm Pma2 R3	–	0.91–3.36	–	–	e:0.18–0.86 h:0.4–2.58
Mixed‐metal Chalcohalides	Sn_2_BiS_2_I_3_^[^ ^23e^ ^]^	orthorhombic	Cmcm	1.41	1.08	direct	n‐type	–
	Pb_2_BiS_2_I_3_ Sn_2_BiS_2_I_3_ Sn_2_BiSI_5_^[^ ^43^ ^]^	orthorhombic orthorhombic monoclinic	Cmcm Cmcm C2/m	1.22–1.92	0.85–1.18	direct	–	–
	Pb_2_SbS_2_I_3_^[^ ^31a^ ^]^	orthorhombic	Cmc2_1_	2.19	1.4	indirect	n‐type	–
	Sn_2_SbS_2_I_3_ Sn_2_SbS_2−_ *_x_* Se*_x_* I_3_^[^ ^44^ ^]^	orthorhombic	Cmcm Cmc2_1_	1.64	–	indirect	–	–
	Sn_2_SbS_2_I_3_^[^ ^14^, ^16^, ^45^ ^]^	orthorhombic	Cmcm Cmc2_1_	1.5–1.8	0.63	indirect	–	–
	CuBiSCl_2_^[^ ^23h^ ^]^	orthorhombic	Cmcm	1.44	1.37	direct	n‐type	e: 0.41 h:1.07
	Cu_3_Bi_6_S_10_I CuBi_2_Se_3_I^[^ ^46^ ^]^	orthorhombic monoclinic	Pnma C2/m	0.68–0.72	1.20–1.25	direct/indirect	p‐type	–
	Al_2_Cu_2_Bi_2_S_3_Cl_8_ AgBiSCl_2_^[^ ^47^ ^]^	hexagonal orthorhombic	P6_3_/m Cmcm	–	1.27 1.08	direct	–	–
	Ag_a_Bi_b_I_‐a+3b‐2x_S_x_^[^ ^23^, ^31^ ^]^	cubic(anti‐perovskite)	Pm3_m	1.85–1.90	–	direct	–	–
Hybrid Organic‐Inorganic Chalcohalides	MA_3_Bi_2_I_9−2x_S_x_^[^ ^48^ ^]^	hexagonal	P6₃/mmc	1.45–2.04	0.83–1.4	direct	n‐type	–
	MASbSI_2_^[^ ^49^ ^]^	cubic	FCC	2.03	–	indirect	n‐type	–
	MABiI_2_S^[^ ^50^ ^]^	Likely orthorhombic or rhombohedral	–	1.52	–	–	–	–

Several special cases among these materials remain unexplained, including those with different stoichiometry and some containing Tellurium (Te).^[^ ^34^, ^35^, ^36^ ^]^ Bi_13_S_18_I_2_, initially reported as Bi_19_I_3_S_27_ by Otto and co‐workers in 1968 and by Miehe and colleagues in 1971 with P6_3_ or P6_3_/m space group,^[^ ^36b,c^ ^]^ presents an intriguing case.^[^ ^36^ ^]^ Groom et al.^[^ ^35a^ ^]^ found out that assuming the partially occupied Bi sites are trivalent (Bi^3^⁺), the charge balance in the average unit cell representation (ACR) structure requires an occupancy of one‐third in P6_3_ or one‐sixth in the 4e position of P6_3_/m. However, crystallographic data from single crystal X‐ray diffraction (XRD) and synchrotron powder XRD (PXRD) suggest a lattice occupancy of ≈24%, a significant deviation from the expected one‐third or one‐sixth.^[^ ^35a^ ^]^ Otto and co‐workers proposed a supercell structure (where a′ = a√13 and c′ = c).^[^ ^51^ ^]^ However, attempts to fit the data were unsuccessful because no extra reflections corresponding to a supercell were observed in any of the diffraction data reported by the same group.^[^ ^36a^ ^]^ Later, Latturner and co‐workers introduced a new hexagonal material named Bi_13_S_18_I_2_, which features pairs of Bi_2_^4+^ ions and is represented by the chemical formula (Bi^3+^)_12_(Bi_2_^4+^)_0.5_(S^2−^)_18_(I^−^)_2_.^[^ ^36a^ ^]^ As shown in Figure 2b, the refinement in space group P6₃ requires splitting the 4e Wyckoff site of P6₃/m into two partially occupied 2a sites, ensuring local charge balance for the ACR‐Bi₁₃S₁₈I₂ structure. Each site is occupied to around one‐third, which allows the Bi^3^⁺/Bi₂⁴⁺ dimers to form while preserving the proposed stoichiometry. Meanwhile, the inset in Figure 2b shows these two bismuth positions lie extremely close (≈0.85 Å), so full occupancy would cause significant steric repulsion.^[^ ^36a^ ^]^ Some chalcohalides containing Te, such as BiTeCl, BiTeBr, and BiTeI exhibit hexagonal (P6_3_mc) for BiTeCl, trigonal (P3m1) for BiTeI, and two‐layer structure ($P\bar{3}m1$) for BiTeBr, respectively.^[^ ^34^ ^]^

#### 3.1.2 Transition/Post‐Transition Metal Chalcohalides

The earliest examples of transition/post‐transition chalcohalide materials date back to the 1960s, with Reuter and Hardel's report on silver bromide (AgBr) and silver sulfide iodide (Ag_3_SI).^[^ ^52^ ^]^ These materials featured an anti‐perovskite structure, where anions and cations occupied opposite positions within the perovskite structure (Figure 2c).^[^ ^24^, ^52^ ^]^ This structure has once again piqued the interest of scientists as it can be linked to the successful attributes of perovskite materials.^[^ ^23j^ ^]^ Caño et al.^[^ ^23j^ ^]^ later fabricated the first PV devices of Ag_3_S(Br_x_I_1‐x_), revealing a cubic structure with $Pm\bar{3}m$ space group for X ≠ 0 and cubic structure with Im3¯m symmetry for X = 0. The α‐Ag₃SI phase remains stable above 569 K and has a high ionic conductivity ≈1.5 S cm⁻¹, which is good for ion transport but undesirable for solar cell applications.^[^ ^23j^ ^]^ In contrast, the β‐Ag₃SI ($\mathrm{Pm}\bar{3}m$) phase is more stable at room temperature and has significantly lower ionic conductivity (approximately two orders of magnitude lower than α‐Ag₃SI).^[^ ^53^ ^]^ This lower ionic conductivity is crucial for maintaining structural stability and minimizing excessive ion migration in solar cells.^[^ ^23j^ ^]^ Excessive ion transport can lead to band bending and hysteresis, ultimately degrading device efficiency over time.^[^ ^54^ ^]^ Additionally, high ionic mobility may induce phase instability and space charge accumulation at interfaces, further limiting charge transport and contributing to material degradation.^[^ ^55^ ^]^

Despite its toxicity, Pb has been widely used in chalcohalide solar cells due to its high efficiency, defect tolerance, and other intriguing properties.^[^ ^31^, ^56^ ^]^ In 2020, Toso et al.^[^ ^23i^ ^]^ synthesized Pb_4_S_3_Br_2_ for the first time using a colloidal method, confirming its Pnma orthorhombic structure through various analyses including 3D‐electron diffraction, XPRD, and HAADF‐STEM. Two years after the initial hypotheses, subsequent research challenged the existing ideas regarding the cubic prototype of Pb_3_S_2_Cl_2_ by revealing a novel monoclinic Cc space group. Figure 2d displays two perspectives of the Cc monoclinic Pb_3_S_2_Cl_2_ structure aligned with the high symmetry axes of the analogous pseudo‐cubic cell, with the top view along the [311] direction and the bottom view along the [111] direction.^[^ ^23^, ^57^ ^]^

In addition to Pb‐ and Ag‐based chalcohalides, computational‐aided methods have been employed to screen potential chalcohalide materials containing other transition metals. Aron Walsh's group identified four promising chalcohalide materials – Cd_4_SF_6_, Cd_5_S_4_Cl_2_, Sn_5_S_4_Cl_2_, and Sn_4_SF_6_ with space groups of $R\bar{3}m$, Cm, Pma2, and R3 and crystal structures of tetragonal, monoclinic, triclinic and octahedral respectively.

#### 3.1.3 Mixed‐Metals Chalcohalides

Mixed‐metal chalcohalides, quaternary chalcohalides comprising combinations of divalent and trivalent cations such as mixing Pb, Sn, Cu, Bi, and Sb, have also attracted attention recently.^[^ ^23^, ^43^, ^44^ ^]^ Kuei‐Fang's group reported Bi_2_CuSe_3_I and Bi_6_Cu_3_S_10_I phases with monoclinic (space group C2/m) and orthorhombic (space group Pnma) crystal structures, respectively.^[^ ^46^ ^]^ The Bi_2_CuSe_3_I is constructed by two distinct layers alternating along the *b*‐axis. Fusing the Bi_(1)_Se_4_I_2_ and Cu_(4)_Se_2_I_2_ polyhedra share edges to form the first layer and the second layer is built up of the edge‐sharing Bi_(2)_Se_5_ square pyramid and an aggregated group of CuSe_4_ tetrahedra. The Bi_6_Cu_3_S_10_I showed a novel 3d‐structure assembled by BiS_5_ square pyramids, BiS_6_ octahedra, BiS_8_ polyhedra, CuS_4_ tetrahedra, and I^−^ anions.^[^ ^46^ ^]^ Very recently, Sun and co‐workers successfully fabricated CuBiSCl_2_, marking the first example of a post‐perovskite structure with Cmcm space group (Figure 2e), where S and Cl shared the anion sites (mixed anion), which is different from the typical anti‐perovskite structure.^[^ ^23^, ^60^ ^]^ This unique characteristic makes CuBiSCl_2_ a promising candidate for solar cells, as split anion materials have demonstrated tunable electronic properties and enhanced stability.^[^ ^61^ ^]^

Kanatzidis and co‐workers synthesized Pb_2_BiS_2_I_3_, Sn_2_BiS_2_I_3_, and Sn_2_BiSI_5_ as statistically disordered materials, each exhibiting distinct crystal structures.^[^ ^13^, ^43^ ^]^ The former two materials show orthorhombic crystal structures with the Cmcm space group, while the latter exhibits a monoclinic structure with the C2/m space group (Figure 2f).^[^ ^43^ ^]^ In an interesting study, Moëlo and co‐workers investigated the crystal structure of Pb_2_SbS_2_I_3_ at room temperature (RT) and low temperature (LT, 100 K).^[^ ^62^ ^]^ It was revealed that Pb_2_SbS_2_I_3_ exhibits an orthorhombic structure with the Cmcm space group at room temperature, and a monoclinic structure with the P2_1_/C space group at 100 K.^[^ ^62^ ^]^ This transition involves the organization of the structure and eliminating mixed sites. In this arrangement, the Pb(1) and Sb(1) elements are positioned alternately along the lengthened parameter c, where c is twice as long as the corresponding parameter a found in the RT structure. Additional experiments conducted at 100 K revealed that the structure solidifies into the P2_1_/c space group, featuring discrete, individualized sites for Pb and Sb. Aron Walsh and co‐workers were further intrigued by the report from Nie et al.^[^ ^23e^ ^]^ on the fabrication of Sn_2_SbS_2_I_3_, because two lone‐pair cations will drive some unusual structure–property relations.^[^ ^16c^ ^]^ Additionally, this material crystallizes in an orthorhombic structure, commonly reported with the Cmcm and Cmc2_1_ space groups, as shown in Figure 2g. First‐principles calculations suggest that the experimentally observed Cmcm structure is a macroscopic average of locally non‐centrosymmetric Cmc2₁ configurations, driven by a second‐order Jahn–Teller distortion of the Sb(III) lone pair. The Cmc2₁ phase is both thermodynamic and dynamic stability, with a formation energy of 35.8 meV per atom lower than that of Cmcm, and no imaginary phonon modes, thereby confirming its stability. These results suggest that the Cmcm phase may transition spontaneously to Cmc2₁ under suitable conditions. Molecular dynamics simulations indicate that Cmc2₁ maintains stability at 300 K, whereas at 500 K, transient Sb atomic hopping indicates a possible transition toward the Cmcm phase, highlighting the temperature‐dependent properties of phase transition.^[^ ^16c^ ^]^ Building on this research, temperature‐induced Sn/Sb cation disorder in Sn₂SbS₂I₃ has been experimentally verified.^[^ ^14b^ ^]^ The transition between the Cmc2₁ and Cmcm phases is accompanied by a noticeable disorder and a bandgap reduction from 1.8 to 1.5 eV upon annealing at 573 K.^[^ ^14b^ ^]^ Monte Carlo simulations further confirm that cation displacement fluctuations play a key role in this phase transition, reinforcing the idea that the observed Cmcm phase may result from local structural distortions. These findings highlight the temperature‐dependent nature of the phase transition and its potential impact on electronic properties. Recently, Schilfgaarde et al.^[^ ^63^ ^]^ synthesized Sn_2_SbS_2−x_Se_x_I_3_, demonstrating a similar crystal structure with band tunability across the S‐to‐Se ratios. Three types of structure were proposed as: 1) the original Cmcm structure with the split Sb reduced from a half‐occupied 8f to a fully occupied 4c site, 2) a P2_1_/c (lower) symmetry structure with staggered occupancy of the 8f Sb site, and 3) the LT‐P2_1_/c configuration of Pb_2_SbS_2_I_3_, utilizing variable‐cell optimization to optimize the atomic and parameters under stable symmetry.^[^ ^44^ ^]^ It is also proposed that the Sn_2_SbS_2_I_3_ shares the same P2_1_/c structure as Pb_2_SbS_2_I_3_ because of the lower ground state energies in low‐temperature P2_1_/c.

The group of Simonov reported that the incorporation of a small amount of sulfide into Ag_a_Bi_b_I_a+3b_, does not reveal different crystal structures for different stoichiometries.^[^ ^31b^ ^]^ AgBiI_4_ and AgBi_2_I_7_ exhibit cubic (Fd3 m) structure, while Ag_3_BiI_6_ belongs to hexagonal structure. Moreover, Ag_3_BiI_6_ and Ag_2_BiI_5_ films exhibited reflections expected for highly crystalline phases with R‐3 m symmetry. In addition, AgBiSCl_2_ and Al_2_Cu_2_Bi_2_S_3_Cl_8_ were evaluated as potential solar absorbers using the Materials Project database and machine learning techniques. Various models, including Neural Networks, Random Forests, and XGBoost, were employed, confirming that AgBiSCl₂ has an orthorhombic crystal structure, while Al_2_Cu_2_Bi_2_S_3_Cl_8_ exhibits a hexagonal structure (Figure 2h).^[^ ^47^, ^64^ ^]^

#### 3.1.4 Hybrid Organic–Inorganic Metal Chalcohalides

Over the past decade, organic–inorganic chalcohalides have been proposed as promising alternatives to lead halide perovskite solar cells.^[^ ^48^, ^49^, ^50^ ^]^ Some hybrid organic–inorganic metal chalcohalides, such as MABiI_2_S and MASbSI_2_, incorporate methylammonium (MA) as a monovalent compact molecular cation. Since these materials are new and lack structural information, the tolerance factor (t) has been used as a useful metric to assess their structural stability. The calculated values of *t* = 0.853 and 0.99 for MABiI_2_S and MASbSI_2_, respectively, suggest that the MABiI_2_S perovskite may prefer an orthorhombic or rhombohedral lattice, while MASbSI_2_ may favor a cubic structure (Figure 2i,j).^[^ ^49^, ^50^, ^65^ ^]^ In a separate investigation, the crystalline structures of CH_3_NH_3_BiSeI_2_ and CH_3_NH_3_BiSI_2_ were studied, revealing a distorted cubic configuration resulting from the incorporation of chalcogen (Figure 2i).^[^ ^58^, ^66^ ^]^ Similar research by Yao and colleagues demonstrated that MA_3_Bi_2_I_9−2x_S_x_ possesses a hexagonal crystal structure within the space group P6_3_/mmc when X = 0.^[^ ^48^ ^]^

### 3.2 Band Position and Bandgap Tuning

The energy gap (*E*_g_), alongside the conduction band minimum (CBM) and valence band maximum (VBM), varies due to factors such as the nature, composition, and stoichiometric proportions of the materials.^[^ ^37^, ^39^, ^67^ ^]^ In theoretical calculations, the Heyd–Scuseria–Ernzerhof (HSE) hybrid functional + spin–orbit coupling (SOC) methods are often employed to estimate accurate optical properties including band gap, dielectric coefficient, and absorption coefficient, which serve as crucial parameters for screening the potential PV absorber candidates.^[^ ^23^, ^42^ ^]^ Chalcohalides exhibit bandgaps ranging from 0.83 to 2.15 eV, offering the opportunity for fabrication of single‐junction or the sub‐cell of tandem solar cells after band structure adjustments (Table 1). Traditional methods for band tuning include but are not limited to: 1) Replacing/ substituting metal cations changes the bond angle and atomic radius. The degree of orbital overlap between the cation and anion varies, changing the energy dispersion of the valence and conduction bands and potentially altering bandgap and carrier transport properties. 2) Substituting chalcogenide and halide anions. For example, replacing sulfur with selenium often leads to a decrease in the E_g_, due to the weaker Se–Se bond strength, increased atomic mass, and slight changes in electronegativity. This leads to a redshift in optical absorption and alters the electronic properties of the material.^[^ ^68^ ^]^ While substituting the halides from chlorine to iodine typically decreases *E_g_* in both Bi‐ and Sb‐based chalcohalides, one DFT calculation proved the trend follows a bandgap narrowing with increasing halogen atomic size, largely due to SOC effects, increased dielectric screening, and enhanced p‐orbital hybridization.^[^ ^69^ ^]^ 3) Adjusting the element's stoichiometry ratio.^[^ ^67^ ^]^

#### 3.2.1 Heavy Pnictogen Chalcohalides

**Figure 3:**
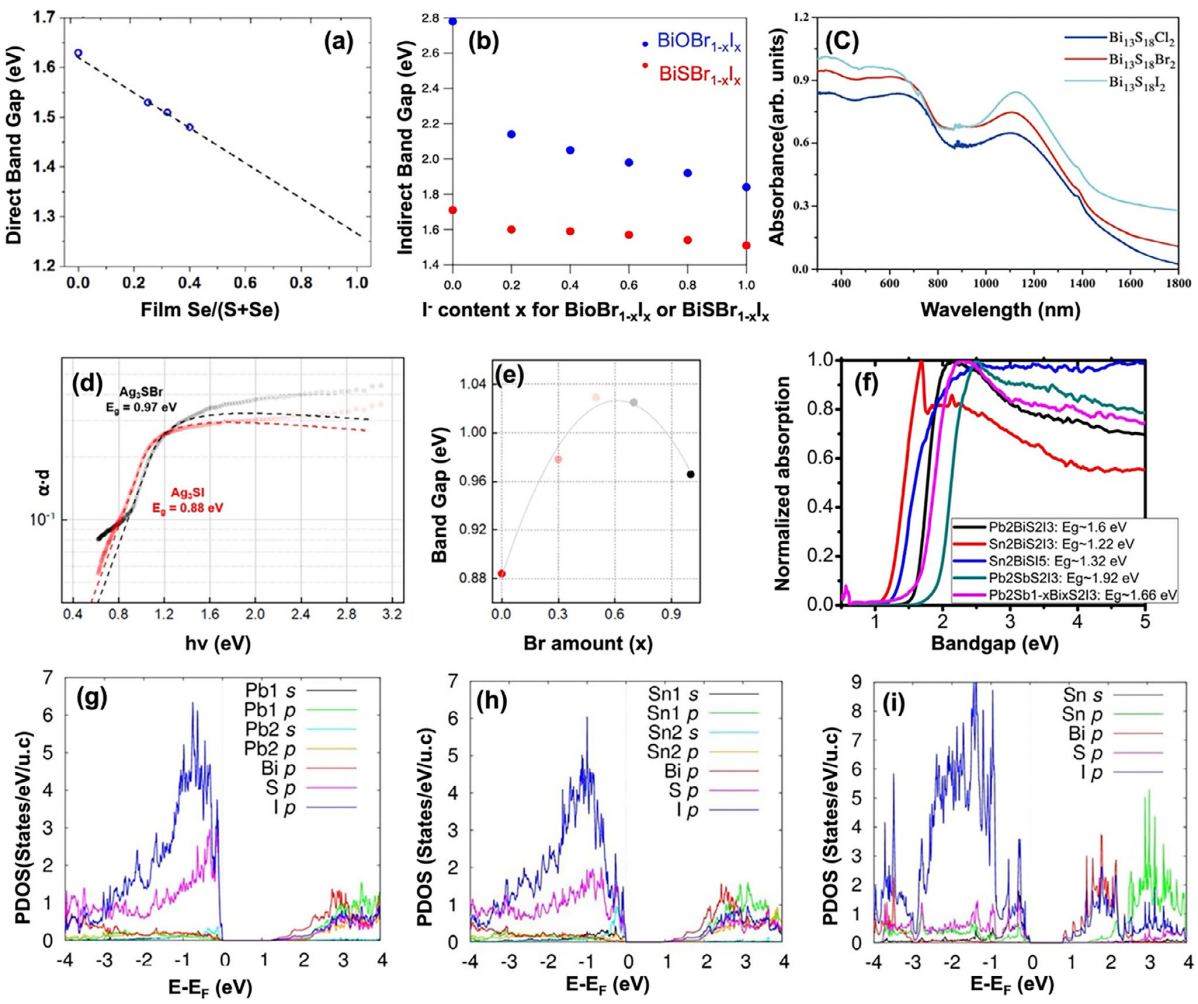
a) The dashed line represents a linear extrapolation of the trend toward BiSeI.^[^ ^23a^ ^]^ b) Indirect bandgap of BiOBr_1−x_I_x_ and BiSBr_1−x_I_x_.^[^ ^15b^ ^]^ c) UV–vis–NIR absorption spectra of Bi_13_S_18_X_2_ powder samples.^[^ ^38^ ^]^ Reproduced with permission.^[^ ^23a^ ^]^ d) Absorption spectra of Ag_3_SBr and Ag_3_SI films by PDS.^[^ ^23j^ ^]^ e) Bandgap as a function of x (Br amount).^[^ ^23j^ ^]^ f) UV/vis absorption spectrum of polycrystalline powder of Pb_2_BiS_2_I_3_, Sn_2_BiS_2_I_3_, Pb_2_SbS_2_I_3_, “Pb_2_Sb_1−_ *_x_* Bi*_x_* S_2_I_3_” (*x* ≈0.4), and Sn_2_BiSI_5_. The bump at ≈1.5 eV in the red spectrum (Sn_2_BiS_2_I_3_) is due to the detector switch which we see frequently in different samples. g–i) projected electronic density of states of g) Pb_2_BiS_2_I_3_ h) Sn_2_BiS_2_I_3_ i) Sn_2_BiSI_5_.^[^ ^43^ ^]^ Copyright 2020, American Chemical Society. Reproduced with permission.^[^ ^15b^ ^]^ Copyright 2016, Nature. Reproduced with permission.^[^ ^38^ ^]^ Copyright 2021, American Physical Society. d,e) Reproduced with permission.^[^ ^23j^ ^]^ Copyright 2024, Royal Society of Chemistry. f–i) Reproduced with permission.^[^ ^43^ ^]^ Copyright 2016, American Chemical Society.

Hahn et al.^[^ ^23a^ ^]^ investigated the bandgap of Bi‐based chalcohalides using UV–vis–NIR diffuse transmittance and reflectance measurements. A linear decrease in bandgap energy *E_g_* from 1.63 to 1.48 eV was observed, corresponding to a transition from a rod‐like to a cubic shape as the Se/(S + Se) ratio varied from 0 to 0.4 in BiS_1‐x_Se_x_I materials. In addition, analysis of pure BiSeI bandgap (1.29 eV) revealed a “bowing” effect rather than a full linear relationship (1.27 eV) (**Figure** **3**a). Theoretical research by Ganose et al.^[^ ^37^ ^]^ demonstrated a reduction in ionization potential and a relative increase in electron affinity for BiSeI compared to BiSI, classifying both materials as n‐type semiconductors. Additionally, DFT calculations were conducted to investigate the factors contributing to low efficiency. The 1D nature of the ribbon structure may partially account for this, as it should be oriented vertically in the device to ensure clear conduction pathways. The large electron affinity of these materials (>4.9 eV), contributes to band misalignments with commonly used electron transport materials, making them unsuitable for carrier transportation. In another work, Abe and co‐workers showed that substituting Br, and O with I and S led to a decrease in bandgap by improving the VBM. Low‐temperature heat treatment of BiOCl with H₂S and H₂Se led to the formation of BiSI and BiSeI. Compared to BiSI, BiSeI showed a more significant redshift in the UV–vis diffuse reflectance spectrum.^[^ ^15b^ ^]^ Additionally, In BiSBr_1‐x_I_x_, the indirect bandgap decreased from 1.71 to 1.51 eV as x increased from 0 to 1.^[^ ^15b^ ^]^ First‐principles calculations combined with absorption spectra (Figure 3b) indicated that: 1) the VBM of BiSBr is mainly comprised of a hybridization of S 3p and Br 4p orbitals, while in BiSI, it is primarily occupied by I 5p orbitals; 2) the 5p orbitals of I contribute more significantly to the valence band formation compared to the 4p orbitals of Br; and 3) the contribution of O 2p to the valence band is minor, while S 3p shows a more pronounced impact.^[^ ^15b^ ^]^ Similar trends were observed in BiTeX, where the band gap was found to increase from 0.33 to 0.72 eV for BiTeI and BiTeCl, demonstrating their potential in applications beyond solar cells.^[^ ^34^ ^]^ Varying stoichiometry was shown to result in different band gaps and band positions in the chalcohalides.^[^ ^34^ ^]^ For example, Li et al.^[^ ^38^ ^]^ reported the first strong cutoff in the visible‐light range as a direct transition for Bi_13_S_18_X_2_, while a second strong cutoff in the NIR range was identified as an indirect transition. The indirect *E_g_* of Bi_13_S_18_Cl_2_, Bi_13_S_18_Br_2_, and Bi_13_S_18_I_2_ were estimated to be 0.76, 0.80, and 0.81 eV, respectively, while the direct bandgaps were 1.34, 1.32, and 1.30 eV. All of these materials were classified as n‐type semiconductors.^[^ ^38^ ^]^ An increase in the atomic radius of the halogen was observed to cause a slight redshift of the absorption edge and an increase in the absorption intensity (Figure 3c).

The Quasiparticle Self‐Consistent GW Theory (QSGW) method was analyzed by Walsh and co‐workers^[^ ^39^ ^]^ estimated the indirect *E_g_* of 2.22, 2.53, and 2.03 eV for SbSI, SbSeI, and SbSBr, respectively. It was noted that this method tends to overestimate results compared to hybrid DFT approaches, primarily due to the exclusion of gap renormalization from electron–phonon interactions and the coupling of electrons and holes in ladder diagrams. Despite the indirect bandgap is generally considered a limitation for PV devices, the case with 1D SbSeI is distinct, as the direct and indirect band edges differ by merely ≈0.2 eV. The minor variation results in a significant optical absorption and reduces the recombination of electron–hole pairs, thanks to the efficient thermalization of carriers throughout momentum space. Subsequently, the ionization potentials were estimated to be between 5.3 and 5.8 eV, highlighting the necessity for suitable electrical contacts in PV applications. From an experimental perspective, Choi et al.^[^ ^70^ ^]^ employed a two‐step spin‐coating method to fabricate Sb(S_1‐x_Se_x_)I. The results showed that as **x** increased, both the CBM and VBM exhibited an upward shift, with the VBM increasing at a more rapid rate. The bandgap gradually decreased from 2.05 to 1.69 eV, demonstrating their potential for tandem PV device fabrication. However, it is essential to carefully consider the suitable charge transport layer and the crystal structure of Se‐rich materials to achieve optimal PV performances.^[^ ^70^ ^]^ In an interesting study, Seok and co‐workers observed that incorporating Bi into SbSI (the resulting material is Sb_0.67_Bi_0.33_SI) led to a reduction of the band gap to 1.62 eV.^[^ ^30^ ^]^ It was found that the VBM and CBM were predominantly influenced by Sb‐5s and Bi‐6p, respectively. The introduction of spin–orbit interaction led to a substantial energy reduction of the Bi‐6p orbitals relative to Sb‐5p orbitals, leading to a significant bandgap reduction upon Bi substitution.

#### 3.2.2 Transition/Post‐Transition Metal Chalcohalides

Recent advancements in the study of Pb‐based chalcohalides, particularly the discovery of novel phases such as Pb_4_S_3_Br_2,_ open promising pathways for materials with tunable *E_g_* for PV and optoelectronic applications.^[^ ^48b^ ^]^ An intriguing observation revealed that the bandgap of Pb_4_S_3_Br_2_ remains unchanged despite variation in size, suggesting that these quantum dots exhibit minimal quantum confinement effects. The indirect bandgap estimated by DFT corresponds with the absence of photoluminescence (PL) signals in the range of 500–1700 nm.^[^ ^48b^ ^]^ The experimentally observed *E_g_* of ≈1.91 eV, with an absorption edge ≈650 nm, coupled with the theoretical support from DFT/PBE calculations (with SOC effects), including spin−orbit coupling (SOC), which yielded a slightly lower *E_g_* of 1.5 eV, emphasizing the role of SOC in determining electronic properties. This discrepancy, often observed when using the PBE functional, highlights the limitations of DFT in fully capturing localized phenomena like hole localization in these complex systems.^[^ ^23^, ^48^, ^71^ ^]^ From their perspective, theoretical insights into Pb_3_S_2_Cl_2_ and Pb_4_S_3_I_2_ materials, with *E_g_* of 2.02 and 1.76 eV, respectively, emphasize the potential of such materials to be fine‐tuned for specific optoelectronic applications, where minor changes in composition could significantly affect performance. Additionally, the investigation into Ag_3_S_x_ materials, particularly into Ag_3_SI with *E_g_* of 0.88 eV and Ag_3_SBr with *E_g_* of 0.97 eV (Figure 3d),^[^ ^23j^ ^]^ presents an intriguing case for understanding how subtle modifications in halide composition such as the incorporation of mixed halides could lead to nonlinear bandgap behaviors. The observed bowing effect in Ag3S(Br_x_I_1−x_) when X = 0.3, 0.5, and 0.7 (Figure 3e) suggests that there are deeper interactions between atomic orbitals at play, reminiscent of what has been observed in other complex solid solutions, such as lead halide perovskites.^[^ ^23j^ ^]^ This phenomenon of bandgap bowing, in our view, opens up a compelling area for further research. Exploring how such effects can be leveraged or controlled in chalcohalide systems could offer new strategies for optimizing materials not only for PV devices but also for broader electronic and photonic technologies.

#### 3.2.3 Mixed‐Metals Chalcohalides

**Figure 4:**
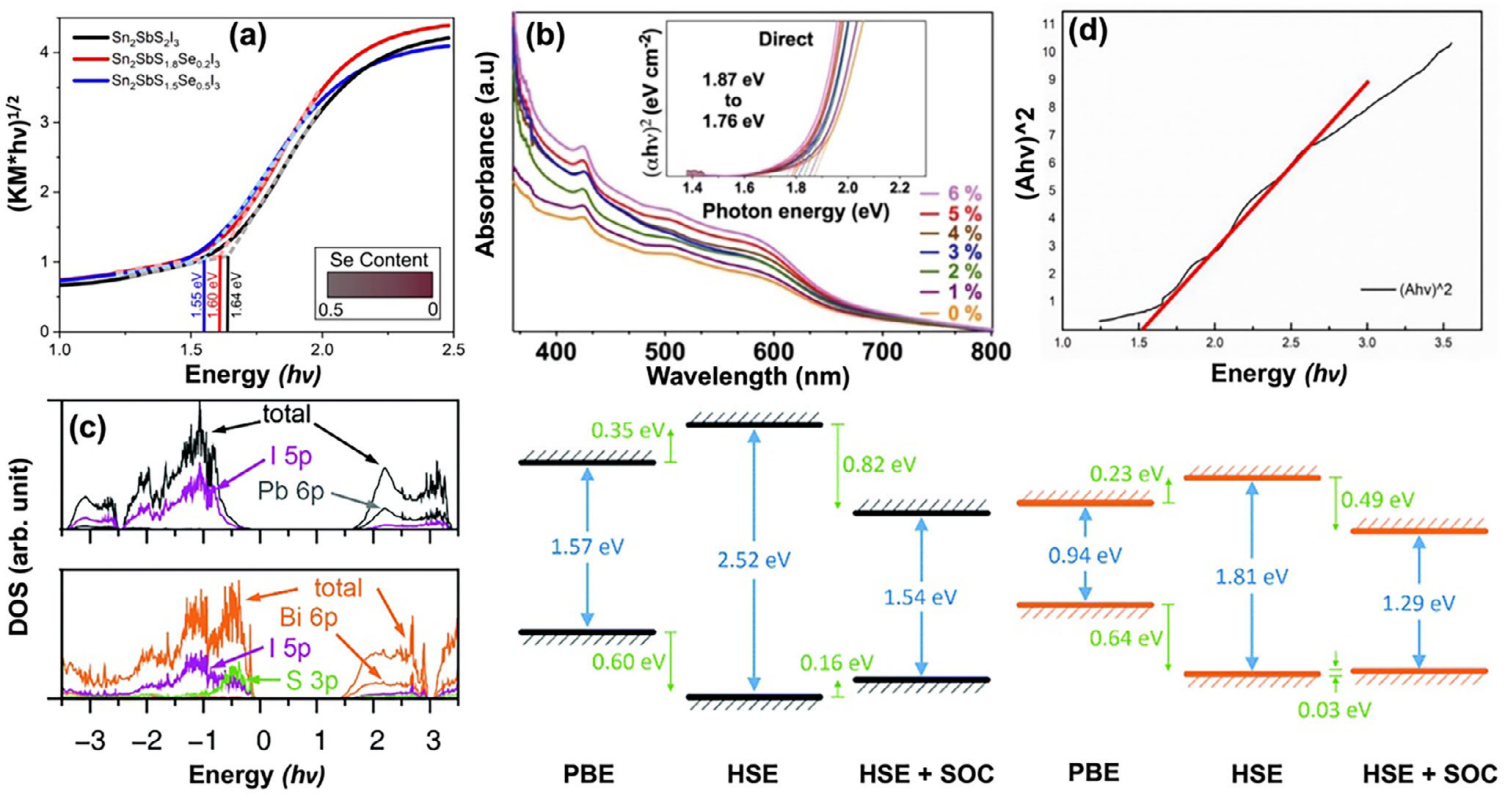
a) Indirect bandgap Tauc plots of solid‐state UV–vis Kubelka–Munk diffuse reflectance spectra for Sn_2_SbS_2−x_Se_x_I_3_.^[^ ^44^ ^]^ b) UV–vis spectra and Tauc plots of Ag_3_BiI_6‐2x_S_x_.^[^ ^31b^ ^]^ c) Density of states of CH_3_NH_3_PbI_3_ and CH_3_NH_3_BiSI_2_ (left) and calculated bandgaps of CH_3_NH_3_PbI_3_ and CH_3_NH_3_BiSeI_2_ (middle, right).^[^ ^58^ ^]^ d) Tauc plots of CH_3_NH_3_BiSI_2_.^[^ ^50^ ^]^ Reproduced with permission.^[^ ^31b^ ^]^ Copyright 2018, Wiley. Reproduced with permission.^[^ ^44^ ^]^ Copyright 2023, Royal Society of Chemistry. Reproduced with permission.^[^ ^58^ ^]^ Copyright 2016, Royal Society of Chemistry. Reproduced with permission.^[^ ^50^ ^]^ Copyright 2019, The Chemical Society of Japan.

Mixed‐metals chalcohalides, another promising material in chalcohalide materials family, exhibit a tunable band gap spanning the entire visible spectrum to the NIR region, showcasing their promise for efficient solar absorption over a wide wavelength (*λ*) region. Islam et al.^[^ ^43^ ^]^ synthesized and measured the bandgaps of Pb_2_BiS_2_I_3_, Sn_2_BiS_2_I_3_, Sn_2_BiSI_5_, and Pb_2_Sb_1−x_Bi_x_S_2_I_3_ (x ≈0.4), all of which displayed direct n‐type bandgaps of 1.60, 1.22, 1.32, and 1.66 eV, respectively (Figure 3f). Notably, compared with the best‐performing Pb_2_SbS_2_I_3_ (2.19 eV) synthesized by Nie's group, substituting Sb with Bi resulted in a significant reduction in the bandgap of 0.59 eV.^[^ ^31a^ ^]^ This reduction is attributed to the greater degree of extension of atomic orbitals and higher SOC of the Bi atom.^[^ ^43^ ^]^ However, in the case of Pb and Sn, the opposite effect was observed, likely due to the high energy of the delocalized lone 5s^2^ pair of electrons on the Sn^2+^ cation. Pb_2_BiS_2_I_3_ exhibited a large hole‐effective mass and small electron‐effective mass due to the flat VB and dispersive CB (Figure 3g). The VB primarily comprises S and I p‐state hybrids with minimal Pb and Bi contributions, while the CBM is primarily composed of Bi p‐states mixed with I and S p‐states. In contrast, Sn_2_BiS_2_I_3_ exhibited a reduced bandgap due to a considerable contribution from the higher energy from 5s states of Sn to the VBM (Figure 3h). The structure of Sn_2_BiSI_5_ introduces a VB with a strong mix of Sn *s*, S *p*, and I *p* states, while its CBM is predominantly characterized by a thorough hybridization of Bi and I p‐states (Figure 3i). Drawing on earlier studies of SbSI, Mori, and co‐workers explored the introduction of Se into Sn_2_SbS_2_I_3_, observing a narrowing of the band gap.^[^ ^44^ ^]^ Specifically, the bandgap of SbS_1.8_Se_0.2_I_3_ was measured as 1.60 eV, while for Sn_2_SbS_1.5_Se_0.5_I_3_, it was found to be 1.55 eV (**Figure** **4**a).

Hsu and co‐workers further reported a new chalcohalide material, namely Bi_2_CuSe_3_I and Bi_6_Cu_3_S_10_I with bandgaps of 0.68 and 0.72 eV, respectively. It should be noted that DFT calculations overestimated the bandgaps to be 1.25 and 1.20 eV mainly due to the lack of spin–orbit interaction (SOI).^[^ ^46^ ^]^ Similar discrepancies between calculated and experimental values have been observed in CuBiSCl_2_ and other computationally screened materials by Ming et al.,^[^ ^23h^ ^]^ where PBE tends to overestimate the *E_g_* compared to most accurate methods like HSE + SOC. In the absence of SOC, the calculation results in a direct bandgap of 1.68 eV, with the CBM and VBM positioned at the Y point. The impact of strong SOC is evident in the comparative results, where the conduction band splits into upper and lower bands, with a separation of ≈0.2 eV. This splitting lowers the CBM by 0.31 eV, reducing the bandgap to 1.37 eV.^[^ ^23h^ ^]^ This emphasizes the importance of SOC in theoretical frameworks concerning chalcohalide materials. The presence of heavy elements such as Pb, Bi, and Sn in chalcohalide materials indicates that SOC significantly influences the electronic structure due to its strong dependence on atomic number (Z^4^). The SOC largely changes the CBM and VBM positions in heavy elements‐based chalcohalides, reduces the bandgap, and modifies the band dispersion. The incorporation of SOC in chalcohalides can result om spin splitting, enhance Rashba effects in non‐centrosymmetric structures, and modify carrier transport characteristics. Consequently, the consideration of SOC is essential for understanding the band structure, defect tolerance, and optoelectronic properties of chalcohalides.

A particularly intriguing finding is that substituting small amounts of S at I sites in Ag_a_Bi_b_I_a+3b−2x_S_x_ resulted in enhanced light absorption and a noticeable redshift in the absorption spectrum.^[^ ^31b^ ^]^ Such a redshift, which is linked to stoichiometric modifications, demonstrates the potential for *E_g_* tuning in these materials, making them more adaptable for optoelectronic applications. Materials with slightly varying stoichiometries displayed similar bandgaps in the range of 1.76 to 1.87 eV, showing a linear drop as X increased (Figure 4b). This trend can be attributed primarily to the elevation of the VBM, improving energy alignment with charge transport layers. More recently, a novel chalcohalide, AgBiSCl_2_, was reported to have a wider bandgap of 2.9 eV. Remarkably, the positioning of Fermi level 1.1 eV above the VBM, but below the midpoint of the bandgap, suggests its intrinsic nature, offering insights into its potential applications in various optoelectronic devices.^[^ ^47a^ ^]^ To further fully exploit the electronic properties of mixed‐metal chalcohalides, a focus on refining the stoichiometry and controlled doping will be key. Such approaches could lead to better charge separation and reduced recombination rates, ultimately enhancing the efficiency of these chalcohalide solar cells.

#### 3.2.4 Hybrid Organic–Inorganic Metal Chalcohalides

To address the toxicity of lead halide perovskite solar cells, Sun et al. used a split‐anion approach to identify the alternatives to CH_3_NH_3_PbI_3_.^[^ ^58^ ^]^ This approach was employed by substituting one I atom per formula with Se or S, while also replacing Pb with Bi to maintain the charge neutrality.^[^ ^58^ ^]^ The bandgaps of CH_3_NH_3_PbSI_2_ and CH_3_NH_3_BiSI_2_, two organic–inorganic chalcohalide materials, were found to be 1.57 and 1.29 eV, respectively, using the PBE function. This approach effectively countered the typical underestimation of standard DFT calculations while addressing the overestimation caused by the exclusion of SOC effects.^[^ ^58^ ^]^ Upon applying the HSE + SOC function, it became evident that the SOC primarily influenced the CBM, leading to a pronounced downshift compared to the VBM. This phenomenon can be attributed to the predominant contribution of Pb or Bi 6P states to the CBM, which results in an overall downward shift of both the VBM and CBM (Figure 4c). Apart from that, the optical absorption and effective mass results indicate that CH_3_NH_3_BiSI_2_ is a strong competitor to CH_3_NH_3_PbI_3_.^[^ ^58^ ^]^ Further experimental insights were gained from investigations of MABiSI_2_ (MBSI), where both the direct and indirect bandgaps were revealed through Tauc plot analysis, establishing a direct bandgap of 1.52 eV (Figure 4d).^[^ ^50^ ^]^ The substitution of Bi with Sb in MBSI resulted in a notable bandgap increase to 2.03 eV while the material retained its n‐type and direct bandgap characteristics.^[^ ^49^, ^50^ ^]^ Additionally, by gradually replacing I with S in (CH₃_3_NH_3_)_3_Bi_2_I through a vapor‐assisted solution process (LP‐VASP), the bandgap was observed to decrease from 2.1 to 1.67 eV, highlighting the potential of elemental substitution to fine‐tune the optical properties of these materials.^[^ ^48a^ ^]^

These findings reveal the wide range of bandgaps exhibited by chalcohalide materials, spanning from 0.33 eV to 2.03 eV, demonstrating diverse potential applications of these materials in single‐junction and tandem solar cells. Low bandgap materials, such as BiS_1−x_Se_x_I (bandgap ranging from 0.33 eV to 1.34 eV), offer significant advantages for use as bottom cells in multi‐junction solar cells, where they can efficiently capture visible‐infrared light. On the other hand, chalcohalide materials with bandgaps ≈1.65 eV to 1.7 eV (e.g., SbSeX) are ideally suited as top cells in tandem solar cells. Higher bandgap materials, such as Pb_2_​BiS_2_​X_2_ (bandgap up to 2.03 eV) can serve effectively as top cells in triple‐junction solar cells, absorbing high‐energy photons. Moreover, some of these materials also show considerable promise in photocatalysis and photoelectrochemical water splitting for hydrogen production, provided their bandgap and energy levels are properly aligned.

### 3.3 Electrical and Dielectric Properties

**Figure 5:**
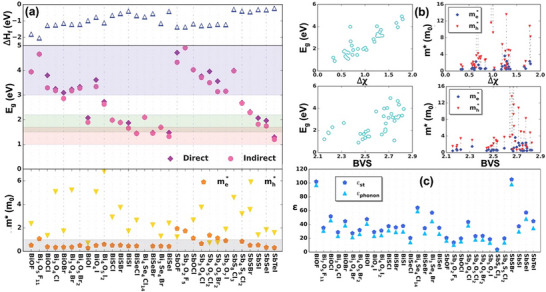
a) formation energies per atom (upper panel), band gaps (middle panel), and effective masses of electron and hole (lower panel) for the 31 Bi and Sb‐based compounds. b) band gaps, hole and electron effective masses as a function of average electronegativity discrepancy between anions and cations for the 31 Bi and Sb‐based compounds; bottom left band gaps, bottom right effective masses of electron and hole as a function of bond valence sum of the cations. c) calculated dielectric constants (ε) of the considered bismuth/antimony oxyhalides and chalcohalides.^[^ ^14a^ ^]^ a–c) Reproduced with permission.^[^ ^14a^ ^]^ Copyright 2018, Nature.

Computational methods have been used to calculate key properties such as formation energy, bandgap, band alignment, effective masses, band valence sum (BVS), and dielectric constants (*ε*) of 31 Bi‐ and Sb‐based chalcohalide materials (**Figure** **5**a–c).^[^ ^14a^ ^]^ Results showed that all materials have negative formation energies, indicating their stability and suitability for experimental synthesis. Most of these chalcohalide materials displayed lower electron effective masses than hole effective masses, which suggests their potential use as hole transport layers to minimize recombination. Interestingly, many of these materials exhibited electron and hole masses close to or below the electron rest mass (*m_0_*), implying promising carrier mobility and high dielectric constants, positioning them as high‐performance PV materials.^[^ ^72^ ^]^ High dielectric constants are essential for improving charge insulation, thus reducing defect charge capture rates, which in turn minimizes scattering, recombination, and deeper defect states, all beneficial for PV applications.^[^ ^73^ ^]^ A detailed study on BiSI and BiSeI revealed that the 1D structure of BiSI nanorods causes anisotropy, with low electron and hole effective masses observed along different crystallographic directions ([010] and [001], respectively).^[^ ^37^ ^]^ Despite the low hole masses, both materials showed small hole diffusion lengths.^[^ ^23b^ ^]^

Electrical conductivity studies on Bi_13_S_18_X_2_ pullet revealed that increasing halide atomic radii led to enhanced electrical conductivity, increasing by approximately one order of magnitude (1.07 × 10^−5^ S cm^−1^ for Cl, 2.58 × 10^−6^ S cm^−1^ for Br, and 8.85 × 10^−7^ S cm^−1^ for I).^[^ ^38^ ^]^ Notably, single‐crystal Bi_13_S_18_I_2_ exhibited significantly higher conductivity than its polycrystalline counterparts, due to reduced resistance at grain boundaries (GBs). Further research by Walsh and co‐workers found isotropic effective masses for SbSI, SbSBr, and SbSeI to be less than 0.65 *m_e_*, supporting high mobility band transport.^[^ ^39^ ^]^

The photoferroic effect, vital for PV applications, helps separate excitons into free carriers through intrinsic crystal polarity, thereby reducing recombination and enhancing open circuit voltage. Among heavy pnictogen chalcohalides, Sb‐based chalcohalides like SbSI and SbSBr exhibit ferroelectric effects, though SbSCl does not.^[^ ^13^, ^74^ ^]^ Temperature‐dependent studies on SbSI and SbSeI revealed distinct ferroelectric and paraelectric phases.^[^ ^48b^ ^]^ Similarly, BiSBr, BiSeI, and BiTeI also exhibit ferroelectric behavior, further demonstrating their potential in optoelectronic applications.

In an interesting screening of four chalcohalides, such as Sn_5_S_4_Cl_2_, Sn_4_SF_6_, Cd_5_S_4_Cl_2_, and Cd_4_SF_6_, DFT calculations showed that materials containing Cd, such as Cd_5_S_4_Cl_2_, and Cd_4_SF_6_, displayed smaller electron effective masses (*m_e_* * = 0.18 and 0.25, respectively) compared to Sn‐based chalcohalides.^[^ ^42^ ^]^ This can be attributed to the higher energy dispersion of s‐orbital electrons in Cd‐based chalcohalides compared to the more directional properties of p‐orbital electrons in Sn‐based chalcohalides. Further, DFT studies on the dielectric constants of CuBiSCl_2_ and MABSI revealed that CuBiSCl_2_ has a static dielectric permittivity (*ε_0_*) of 33.3, mainly due to ionic contributions, while its high‐frequency permittivity (*ε_∞_*) is significantly lower at 8.7, indicating reduced influence from ionic nature at higher frequencies.^[^ ^23^, ^65^ ^]^ Although these values are lower than those of lead halide perovskite, they are still three times higher than those of Si and Cu_2_ZnSnS_4_ solar absorbers, making CuBiSCl_2_ a competitive material for solar cell applications.

### 3.4 Defect Properties

**Figure 6:**
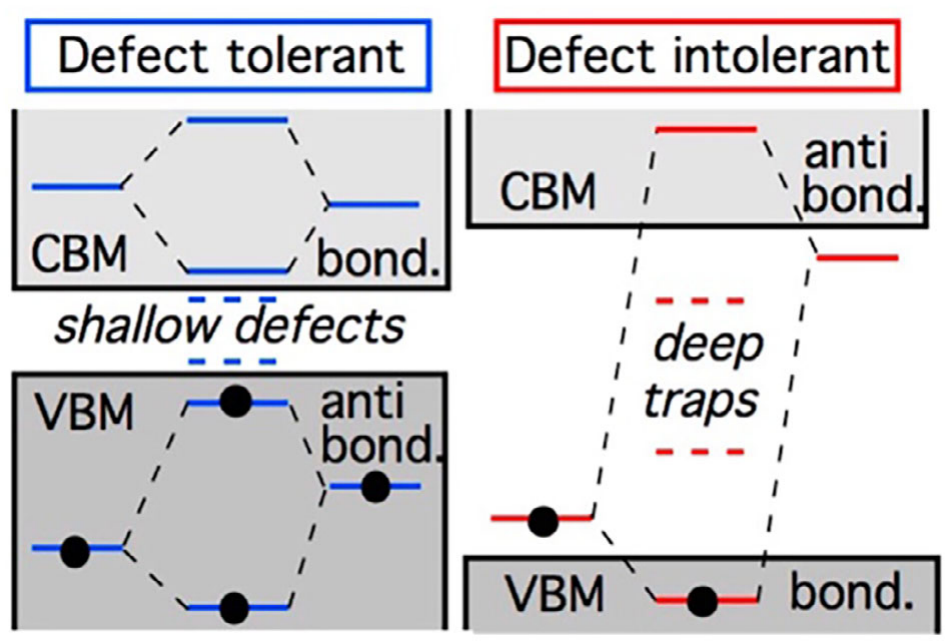
Defect tolerance versus defect intolerance.^[^ ^75c^ ^]^ Reproduced with permission.^[^ ^75c^ ^]^ Copyright 2014, America Society Chemistry.

Defect tolerance is a key factor influencing the carrier density and mobility of materials.^[^ ^75^ ^]^ Materials with strong defect tolerance often demonstrate high PCE, even in the presence of various defect types, such as point defects and GBs.^[^ ^75^, ^76^ ^]^ According to Andriy Zakutayev from NREL,^[^ ^75c^ ^]^ semiconductors with antibonding states at the top of the VB tend to be defects tolerant (**Figure** **6**). The involvement of a cation's s‐orbital in antibonding states leads to an asymmetric electron density distribution, which helps stabilize the structure.^[^ ^23^, ^77^ ^]^ This hybridization of anion p orbitals and cation s orbitals enhances VB dispersion, reducing electron effective masses and increasing carrier mobility.^[^ ^23^, ^78^ ^]^ This relationship between reduced effective electron mass and increased mobility plays a crucial role in defect tolerance and improved PCE.

Materials with an ns^2^ electronic configuration further contribute to this defect tolerance by exhibiting a high dielectric constant, low carrier effective masses, and an antibonding nature of the VB, all of which promote the formation of shallow defects.^[^ ^72^, ^75^ ^]^ These shallow defects are less likely to trap charge carriers, resulting in enhanced conductivity and efficiency in solar cells.^[^ ^13a^ ^]^ Chalcohalide materials containing metal cations such as Bi, Sb, Cu, Pb, and Sn, which possess ns^2^ electronic configurations, are promising candidates to replace Pb‐based halide perovskite solar absorbers due to their similar defect‐tolerant properties (Figure 6).^[^ ^13^, ^23^ ^]^

In summary, chalcohalides hold strong potential as next‐generation absorber materials and viable alternatives to Pb‐based halide perovskite solar cells, mainly due to their remarkable crystal structure and optoelectronic properties. Most lab‐scale polycrystalline thin film devices such as perovskites and chalcohalides, are synthesized via low‐temperature solution processes. While this approach is straightforward, low processing temperatures and complex evaporation steps can lead to a high density of GBs, thereby negatively impacting device performance. Therefore, selecting defect‐tolerant materials is crucial for advancing thin film PV devices. Beyond defect tolerance, some Bi‐ and Sb‐based chalcohalides exhibit effective masses only slightly higher than those of Pb‐based halide perovskites, which remain favorable for carrier transport.^[^ ^24a^ ^]^ Moreover, their high absorption coefficients enable the use of thinner films, broadening their potential applications. Their wide range bandgap tunability further enhances their suitability for tandem/multijunction solar cells. However, studies on defect states in chalcohalides remain limited. Systematic computational analyses on defect states and their formation energies could provide insights for targeted passivation strategies, facilitating the fabrication of larger grain sizes and improving device performance. In addition, a deeper understanding of these materials will be essential for designing more efficient solar cell architecture. The following section explores device structures and their working principles in more detail.

## 4 Device Structure and Their Working Principles

**Figure 7:**
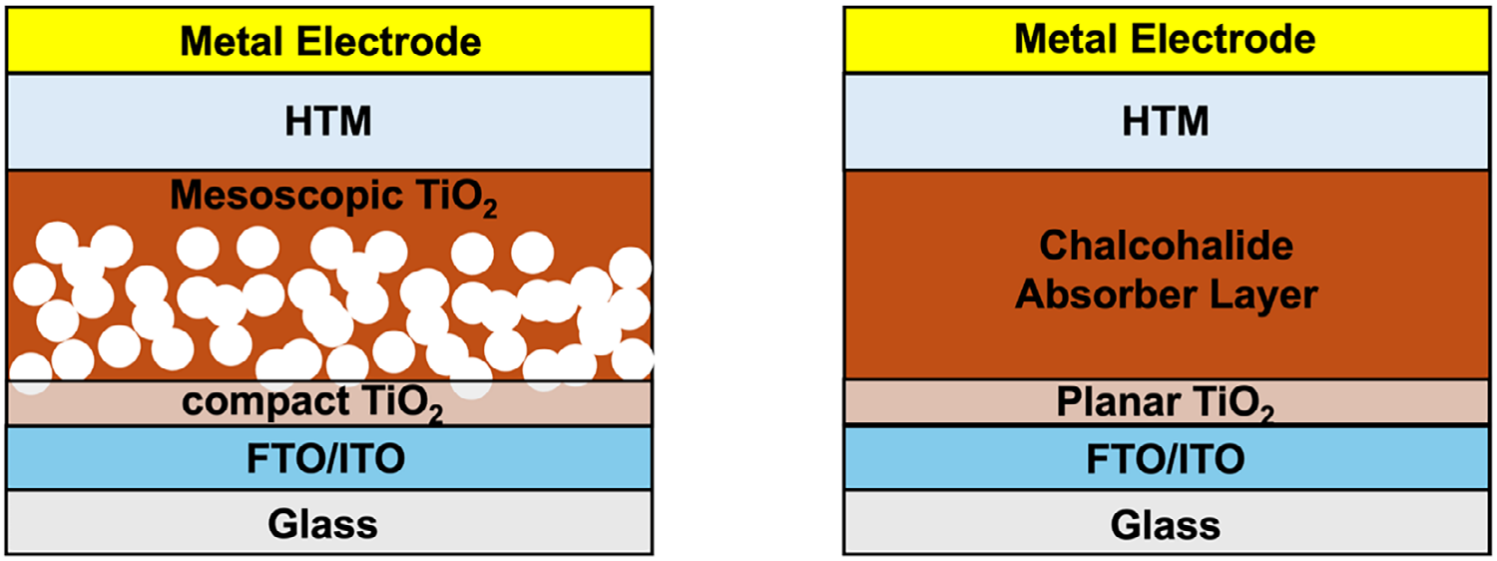
Mesoscopic structured solar cell versus n–i–p planar structured solar cell.

Chalcohalide solar cells typically adopt two primary device structures: planar and mesoscopic.^[^ ^67^ ^]^ Both structures incorporate key components such as an absorber layer, electron transport layer (ETL), hole transport layer (HTL), transparent conducting oxides (TCO), and metal contacts. The only difference in mesoscopic structure compared to the planer structure is the use of HTL and ETL scaffolds.^[^ ^13^, ^23^, ^24^, ^34^, ^67^ ^]^ The planar structures are further classified as conventional (p–i–n) and inverted (n–i–p) configurations, depending on whether the HTL or ETL is adjacent to the light source.^[^ ^67^ ^]^ However, the most chalcohalide solar cells have been fabricated in the conventional n–i–p structure. **Figure** **7** illustrates the schematic representation of mesoscopic and n–i–p planar structured devices, with the mesoscopic structure shown on the left and the planar structure on the right. In an n–i–p device, incident light passes via the TCO, usually fluorine‐doped tin oxide (FTO) or indium tin oxide (ITO), from the glass substrate. The chalcohalide absorber layer on the TCO absorbs the light to produce electron–hole pairs. The electrons are excited to the conduction band, while holes remain in the valence band. Driven by the built‐in electric field or favorable band alignment at the interfaces, electrons travel toward the ETL, generally consisting of materials such as TiO_2_ or SnO_2_, and are subsequently collected by the TCO electrode. Simultaneously, holes diffuse toward HTL, such as CuSCN, and eventually are collected by the metal contact (e.g., Au or Ag).

Among these, mesoscopic structures have seen wider adoption due to their high porosity and large surface area (up to 1000 m^2^ g^−1^), which benefits the absorber layer with short exciton diffusion lengths by facilitating efficient carrier separation and transport.^[^ ^79^ ^]^ In n–i–p mesoscopic solar cells, for example, electrons are transported through both the absorber layer and the TiO_2_ scaffold before reaching the FTO conducting layer.^[^ ^67^ ^]^ TiO_2_ is favored as an ETL material due to its excellent electron mobility, chemical and thermal stability, and favorable band alignment with a variety of semiconductors.^[^ ^23^, ^79^, ^80^ ^]^ Typically, both the blocking layer (BL‐TiO_2_) and mesoporous TiO_2_ (mp‐TiO_2_) are employed, with BL‐TiO_2_ preventing charge recombination by blocking electrons backflow, while mp‐TiO_2_ serves as a scaffold for the chalcohalide materials and facilitate efficient electron transport.^[^ ^67^ ^]^ Alternate mesoscopic materials such as zinc oxide (ZnO) and zirconium dioxide (ZrO_2_) have also been utilized.^[^ ^40^, ^81^ ^]^ The HTL plays an equally important role in effectively transporting holes from the chalcohalide absorber to the electrode, thereby improving solar cell efficiency.^[^ ^70^, ^82^ ^]^

For heavy pnictogen and metal chalcohalide solar cells, polymers such as poly(3‐hexylthiophene) (P3HT) and poly[2,6‐(4,4‐bis‐(2‐ethylhexyl)‐4H‐cyclopenta[2,1‐b;3,4‐b′]dithiophene)‐alt‐4,7‐(2,1,3‐benzothiadiazole)] (PCPDTBT), as well as Poly(3,4‐ethylenedioxythiophene) polystyrene sulfonate (PEDOT:PSS), are commonly employed as HTLs.^[^ ^23^, ^83^ ^]^ In hybrid organic–inorganic chalcohalide solar cells, 2,2′,7,7′‐Tetrakis [N,N‐di(4‐me‐thoxyphenyl) amino]‐9,9′‐spirobifluorene (Spiro‐OMeTAD) has emerged as preferred HTL material.^[^ ^48^, ^50^, ^65^ ^]^ The narrow bandgap of PCPDTBT HTL has been shown to absorb long wavelengths and contribute to additional PCE,^[^ ^23d^ ^]^ which opens up further opportunities for studying HTL materials. Highly conductive precious metals like Au, Ag, and Pt are typically used as electrodes, but there is potential for cost reduction through the use of alternatives such as single‐layer graphene (SLG) and Sn, which show promise for replacing noble metals.^[^ ^23^, ^40^ ^]^

In comparison, planar structures have demonstrated similar transport rates but higher diffusion lengths, increased recombination resistance, and lower recombination rates, resulting in higher PCEs in perovskite solar cells.^[^ ^67^, ^84^ ^]^ These advantages could be used to further improve the efficiency of chalcohalide solar cells in the future. In planer nanocrystal solar cells, the absorber layer is directly sandwiched between the ETL and HTL, which simplifies electron–hole separation and enhances transport efficiency. ETL materials such as tin oxide (SnO_2_) and aluminum‐doped zinc oxide (AZO) have already been employed in Bi‐ and Sb‐based solar cells,^[^ ^23^, ^38^ ^]^ while HTLs such as poly(9,9‐di‐noctylfluorenyl‐2,7‐diyl) (F8) have been used in BiSI solar cells.^[^ ^38^ ^]^ The emergence of planar structures in chalcohalide‐based heterojunction solar cells further highlights the potential for advancements, as demonstrated by recent work on combining p‐CuSCN with n‐BiSI and p‐CdS with n‐SbSeI to fabricate the chalcohalide solar cell.^[^ ^40^, ^85^ ^]^

To enhance PCE in both mesoscopic and planar device structures, it is essential to first understand the crystal structure and physical properties of chalcohalides. Although many chalcohalides exhibit suitable band energies, their open‐circuit voltage remains quite low. This can be attributed to two main factors: i) band mismatch between the absorber and charge transport layers, and ii) energy‐level misalignment compared to traditional halide perovskites. Although employing well‐established halide perovskite‐based charge transport layers provides a temporary solution; designing a new charge transport layer specifically for chalcohalides is the key to achieving comparable device efficiencies to other PV technologies. For example, increasing the mesopore size of mp‐TiO_2_ has been shown to improve charge transfer efficiency by allowing full penetration of the absorber layer and HTL.^[^ ^30^ ^]^ Doping PC_60_ BM into PCPDTBT has further bridged an electron channel between mp‐TiO_2_ and PCPDTBT. It is evident that optimizing the absorber layer and HTL/ETL boundary direction is key to enhancing carrier mobility, and the continuous phase morphology of HTL is critical to high device performance.^[^ ^40^ ^]^

Another issue lies in the crystal structure. The widely known chalcohalide typically exhibits 1D nanorods and 2D nanosheet structures, resulting in a high density of GBs and defect sites. Unlike cubic halide perovskites, which can achieve a highly ordered structure, chalcohalide materials face challenges with directional alignment, leading to increased non‐radiative recombination losses. An arrangement of 1D and 2D chalcohalide materials that is more vertically aligned may enhance charge carrier transport efficiency. The first report on BiSI solar cells estimated a hole diffusion length of ≈5 × 10⁻⁶ cm, emphasizing the need for precise control of film thickness to optimize light absorption and charge extraction in spin‐coating methods.

To further understand the device structure and thereby improve the device efficiency, several other properties, including dielectric constant, effective masses, absorption coefficient, and charge carrier concentration, should also be studied beyond the crystal structure. Chalcohalide research is currently in its early stages. Therefore, drawing inspiration from the development roadmap of perovskite solar cells, the immediate priority is to improve chalcohalide film quality by achieving smooth, compact, and polycrystalline microstructures. Simultaneously, both experimental and theoretical investigations should identify the most optimal chalcohalide compositions and stoichiometric ratios. Subsequently, post‐treatment strategies, including passivation techniques, can be utilized to further enhance grain quality and reduce defect densities in chalcohalide materials.^[^ ^86^ ^]^ Various strategies have been explored including but not limited to interfacial engineering, defect passivation, and heterostructure engineering.

In the pursuit of improving chalcohalide solar cell efficiency, lessons learned from perovskite solar cells, particularly regarding charge‐selective contacts and interfacial engineering, should be applied. The optimization of interfacial morphologies to reduce defect densities is essential for improving charge collection efficiency. Continued research will be necessary to understand the intrinsic properties of bulk defects and to improve carrier extraction efficiencies from the ETL and HTLs.

## 5 Fabrication Methods for Chalcohalide Solar Cells

**Figure 8:**
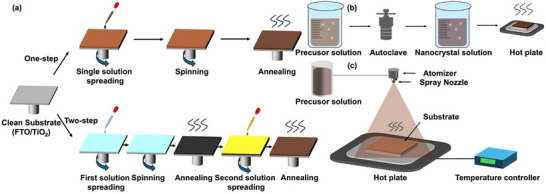
Schematic illustration of some solution processes for chalcohalide solar cell fabrication. a) One‐step and two‐step spin‐coating method, b) Chemical deposition method, and c) Spray‐pyrolysis method.

The fabrication of chalcohalide solar cells can be broadly classified into two main methodologies: solution processes (**Figure** **8**) and vacuum processes. Each technique has distinct advantages and limitations, making them complementary to one another based on the desired application and material properties of the solar cells.

### 5.1 Solution Processes

The majority of the chalcohalide solar cells have been fabricated by solution process due to their cost‐effectiveness, versatility, and suitability for large‐scale production. Among these chalcohalides, heavy pnictogen chalcohalides (MXY, where M = Bi or Sb; X = S, Se, or Te; and Y = Cl, Br, or I) have attracted significant attention in the field of solar cells. Their popularity is due to their appealing ferroelectric behavior, defect‐tolerant nature, low electron/hole effective masses, and a tunable bandgap ranging from 0.7–2.0 eV.^[^ ^24^, ^40^ ^]^ These remarkable properties make them suitable for use as both absorber for single‐junction solar cells, as well as the top/bottom cell absorber in tandem solar cells. Representative chalcohalide solar cell compositions, synthesis methods, device structures, efficiencies, and stabilities achieved are summarized in **Table** **2**.

**Table 2:** Representative chalcohalide solar cells compositions, synthesis method used, device structure adopted, efficiency and stability achieved.

Type	Composition	Synthesis method	Device structure	Bandgap (E_g_) [eV]	PCE/J_sc_/V_oc_/FF	Long‐term stability
Heavy Pnictogen Chalcohalides	BiSI^[^ ^23b^ ^]^	Spray pyrolysis	FTO/n‐BiSI in NaI + acetonitrile electrolyte Reference electrode: Ag/AgCl	Direct 1.63 Indirect 1.57	IPCE ≈ 38% at 500 nm at + 0.4 V vs Ag/AgCl J_sc_ = 5 mA cm^−2^ at 0.4 vs Ag/AgCl Onset potential = −0.25 V vs Ag/AgCl	Remain stable for up to 5 min and degrade to one‐third of performance after 10 min.
	BiS_1−x_Se_x_I^[^ ^23a^ ^]^	Spray pyrolysis	FTO/n‐BiSI/p‐CuSCN/FTO‐Pt	1.63 X = 0 1.48 X = 0.4	PCE = 0.012% J_sc_ = 70 µA cm^−2^ V_oc_ = 0.39 V FF = 40% X = 0	Solid state solar cell: Robust
	BiSI^[^ ^15^, ^37^ ^]^	Soft chemical method	FTO/BiSI in NaI + acetonitrile electrolyte Reference electrode: Ag/AgCl	Indirect 1.51	IPCE: 64% at 700 nm at + 0.2 V vs Ag/AgCl J_sc_ ≈ 3.2 mA cm^−2^	–
	BiSI^[^ ^83b^ ^]^	Two step spin coating	FTO/TiO_2_/BiSI/P3HT/Au	1.61	J_sc_ = 0.1 mA cm^−2^ V_oc_ = 0.4 mV	–
	BiSI^[^ ^87^ ^]^	Spin coating	Glass/FTO/SnO_2_/BiSI/F8/Au	Direct 1.57	PCE = 1.32% J_sc_ = 8.44 mA cm^−2^ V_oc_ = 0.445 v FF = 35.14%	–
	Bi_13_S_18_I_2_^[^ ^23c^ ^]^	Solvothermal	Anode: FTO/ pl‐TiO_2_/mp‐TiO_2_/Bi_13_S_18_I_2_ Counter electrode: FTO/Pt Electrolyte: LiI, I_2_, 1‐butyl‐3‐n‐propylimidazolium iodide, 4‐tertbutylpyridine, and guanidine thiocyanate in a mixed solvent of acetonitrile and valeronitrile (Vol.‐Vol. = 85:15).	0.75 Indirect	PCE = 0.85% J_sc_ = 3.82 mA cm^−2^ V_oc_ = 0.58 V FF = 38.32%	Relatively high repeatability originates from the high stability of Bi_13_S_18_I_2_.
	Bi_13_S_18_I_2_^[^ ^38^ ^]^	Solvothermal	Anode: FTO/pl‐TiO_2_/mp‐TiO_2_/Bi_13_S_18_I_2_/elec./Pt Counter electrode: FTO/Pt Reference electrode: Ag/AgCl Electrolyte: LiI, I_2_, 1‐butyl‐3‐n‐propylimidazolium iodide, 4‐tert‐butylpyridine, and guanidine thiocyanate in a mixed solvent of acetonitrile and valeronitrile (Vol.‐Vol. = 85:15).	Indirect 0.81	PCE = 0.75%	–
	BiSI^[^ ^85^ ^]^	Solvothermal	ITO/p‐CuSCN/n‐BiSI/W	Indirect 1.6	PCE = 0.66% J_sc_ = 2.73 mA cm^−2^ V_oc_ = 0.46 V FF = 53.81%	–
	BiSCl^[^ ^88^ ^]^	Solvothermal	FTO/TiO_2_/BiSCl/ (I^3−^/I^−^)/Pt Reference electrode: Ag/AgCl	Direct 1.96	PCE = 1.36% J_sc_ = 9.87 mA cm^−2^ V_oc_ = 0.54 V FF = 25.5%	High stability But relatively low thermal stability
	Bi_13_S_18_Br_2_^[^ ^38^ ^]^	Solvothermal	Anode: FTO/pl‐TiO_2_/mp‐TiO_2_/Bi_13_S_18_Br_2_/elec./Pt The same electrolyte and counter electrode in Bi_13_S_18_I_2_^[^ ^38^ ^]^	indirect 0.80	PCE = 1.12%	–
	Bi_13_S_18_Cl_2_^[^ ^38^ ^]^	Solvothermal	Anode: FTO/pl‐TiO_2_/mp‐TiO_2_/Bi_13_S_18_Cl_2_/elec./Pt The same electrolyte and counter electrode in Bi_13_S_18_I_2_^[^ ^38^ ^]^	Indirect 0.76	PCE = 0.91%	–
	BiEX (E = S, Se and X = Cl, Br, I)^[^ ^69^ ^]^	Hot‐co‐injection	ITO/BiSI/(I^3−^/I^−^)/Pt Reference electrode: Ag/AgCl	Direct BiSCl:2.55 BiSBr:2.25 BiSI:1.60 Indirect BiSCl:2.05 BiSBr:1.95 BiSI:1.50	BiSI: IPCE in the vis spectrum is above 10% J_sc_ = 0.2 mA cm^−2^ V_oc_ = 0.25V	BiSBr: Slightly oxidation after 1‐month ambient condition; above 250 °C, turned into BiOBr BiSI: No degradation upon intense irradiation (100 mW cm^−2^) in the corrosive environment of an electrolytic solution containing the iodine/iodide redox couple.
	SbSI^[^ ^23d^ ^]^	Spin coating	FTO/TiO_2_‐BL/mp‐TiO_2_/SbSI/PCPDTBT/Au	2.15	PCE = 3.05% J_sc_ = 9.11 mA cm^−2^ V_oc_ = 0.58 V FF = 57.7%	93% of the initial PCEs after storing for 15 days under humidity ambient
	SbSI^[^ ^83a^ ^]^	Two‐step spin coating	FTO/TiO_2_/SbSI/P3HT/Au	1.96	PCE = 0.93% J_sc_ = 5.45 mA cm^−2^ V_oc_ = 0.548 V FF = 31%	90% of initial PCEs after 30 days at 40% humidity.
	SbSI^[^ ^81^ ^]^	Sonication‐heating	FTO/pl‐TiO_2_/mp‐TiO_2_/SbSI/ZrO_2_/C	1.88	PCE = 0.035% J_sc_ = 0.4 mA cm^−2^ V_oc_ = 290 mV FF = 0.31%	Stable after stored for 3 months
	Sb_0.67_Bi_0.33_SI^[^ ^30^ ^]^	Chemical bath deposition (CBD) + Spin coating	FTO/TiO_2_‐BL/mp‐TiO_2_/SbSI/PCPDTBT:PC_60_BM (10:5)/PEDOT:PSS/Au	1.62	PCE = 4.07% J_sc_ = 14.54 mA cm^−2^ V_oc_ = 0.53 V FF = 52.8%	92% of initial PCEs under ambient conditions of 60% humidity over 360 h, 93% of initial PCEs after 1 sun illumination for 1254 min. and 92% after storage at 85 °C in air for 360 h.
	SbSI^[^ ^89^ ^]^	CBD + Spin coating	FTO/TiO_2_‐BL/mp‐TiO_2_/SbSI/PCPDTBT/Au	1.76	PCE = 3.62% J_sc_ = 9.26 mA cm^−2^ V_oc_ = 0.6 V FF = 65.2%	92% of the initial PCE after one month in three types of environments (60% humidity/85 °C + 40% humidity/ without UV block filter in standard test condition (STC) for 1950 min. at 25 °C)
	Sb_2_S_3_‐SbSI^[^ ^89^ ^]^	CBD + Spin coating	FTO/TiO_2_‐BL/mp‐TiO_2_/Sb_2_S_3_/SbSI/HTM[PCPDTBT/poly (3,4‐ethylenedioxythiophene) doped with poly(4‐styrenesulfonate)]/Au	–	PCE = 6.08% J_sc_ = 14.92 mA cm^−2^, V_oc_ = 0.62 V FF = 66%	–
	SbSI‐PAN^[^ ^82^ ^]^	Sonochemical synthesis	Glass/ITO/TiO_2_ nanoparticles/SbSI‐PAN nanocomposite/P3HT/Au	1.76	Sb_2_S_3_(60 wt.%)‐PAN(40 wt.%): J_sc_ = 1.84 µA cm^−2^ V_oc_ = 93.7 mV	–
	SbSeI^[^ ^90^ ^]^	Spin‐coating	FTO/TiO_2_‐BL/mp‐TiO_2_/SbSeI/PCPDTBT/ PEDOT:PSS/Au	1.67	PCE = 4.1% J_sc_ = 14.8 mA cm^−2^ V_oc_ = 473.0 mV FF = 58.7%	90% of initial PCE after illuminating under AM 1.5G (100 mW cm^−2^) for 2321 min. Good stability regardless of humidity, temperature, and illumination.
	SbSeI^[^ ^40^ ^]^	Co‐evaporation + high pressure halogenation	SLG/Mo/SbSeI/CdS/ZnO/ITO	1.78	PCE = 0.81% J_sc_ ≈ 6 mA cm^−2^ V_oc_ = 350 mV FF ≈ 32%	–
	SbSeBr^[^ ^40^ ^]^	Co‐evaporation + high pressure halogenation	SLG/Mo/SbSeBr/CdS/ZnO/ITO/Ag	–	PCE = 0.6% J_sc_ = 2.89 mA cm^−2^ V_oc_ = 591.7 mV FF = 34.2%	–
Transition and Post‐Transition Metal Chalcohalides	Pb_3_S_2_Cl_2_, Pb_4_S_3_Br_2_, Pb_4_S_3_I_2_^[^ ^23i^ ^]^	Spin coating	ITO/AZO/Pb_4_S_3_Br_2_/MoO_x_/Au	Pb_4_S_3_Br_2_ 1.98	Pb_4_S_3_Br_2_ PCE = 0.21% J_sc_ = 1.2 mA cm^−2^ V_oc_ = 0.57 V	Good stability under ambient atmosphere, retaining 60% of the PCE after more than 2 months of storage
	Ag_3_SI^[^ ^23j^ ^]^	Spin coating	FTO/TiO_2_/Ag_3_SI/P3HT/Au	0.88	PCE = 0.005% V_oc_ = 20–40 mV	–
	Ag_3_SBr^[^ ^23j^ ^]^	Spin coating	FTO/TiO_2_/Ag_3_SBr/P3HT/Au	0.97	PCE = 0.015% V_oc_ = 100–150 mV	–
Mixed‐Metal Chalcohalides	Pb_2_SbS_2_I_3_^[^ ^31a^ ^]^	CBD + spin coating	FTO/TiO_2_‐BL/mp‐TiO_2_/Pb_2_SbS_2_I_3_/ PCPDTBT/Au	2.19	PCE = 3.12% J_sc_ = 8.79 mA cm^−2^ V_oc_ = 0.61 V FF = 58.2%	90% of the initial PCEs in 30 days under a humidity environment.
	Ag_3_BiI_6‐2x_S_x_^[^ ^31b^ ^]^	Spin coating	FTO/c‐TiO_2_/m‐TiO_2_/ Ag_3_BiI_5.92_S_0.04_/PTAA/Au	Ag_3_BiI_5.92_S_0.04_ 1.8	PCE = 5.44% J_sc_ = 14.6 mA cm^−2^ V_oc_ = 569 mV FF = 66%	90% of the initial PCEs after 45 days of storage under ambient conditions.
	Sn_2_SbS_2_I_3_^[^ ^23e^ ^]^	Spin coating	FTO/TiO_2_‐BL/mp‐TiO_2_/Sn_2_SbS_2_I_3_/ PCPDTBT/PEDOT:PSS/Au	1.41	PCE = 4.04% J_sc_ = 16.1 mA cm^−2^ V_oc_ = 0.44 V FF = 57%	Exhibited good stabilities at 80% relative humidity, 85 ^o^C in air, and under illumination, respectively.
	CuBiSCl_2_^[^ ^23h^ ^]^	Solid state reaction	FTO/BL‐TiO_2_/mp‐TiO_2_/CuBiSCl_2_/Sn	1.37	PCE = 1.0% J_sc_ = 1.38 mA cm^−2^ V_oc_ = 1.09 V FF = 66%	Up to 300 °C thermal stability 25 days of storage at ambient conditions with 60% relative humidity.
Hybrid Organic–Inorganic Chalcohalides	MASbSI_2_^[^ ^49^ ^]^	Successive ionic layer adsorption and reaction (SILAR)	FTO/TiO_2_‐BL/mp‐TiO_2_/MASbSI_2_/ PCPDTBT/ PEDOT:PSS/Au	2.03	PCE = 3.08% J_sc_ = 8.12 mA cm^−2^ V_oc_ = 0.65 V FF = 58.5%	90% of the initial PCEs for up to 15 days under 60% humidity
	MABiI_2_S^[^ ^50^ ^]^	Two‐step solid‐state reaction	Glass/FTO/c‐TiO_2_/mp‐TiO_2_/ MABiI_2_S/Spiro‐OMeTAD/Au	1.52	PCE = 0.13% J_sc_ = 1.96 mA cm^−2^ V_oc_ = 0.22 V FF = 30%	–
	MA_3_Bi_2_I_9−2x_S_x_^[^ ^48a^ ^]^	Spin Coating	FTO/c‐TiO_2_/mp‐TiO_2_/MA_3_Bi_2_I_9‐2x_S_x_/ Spiro‐OMeTAD/Au	MA_3_Bi_2_I_9−2x_S_x_: 1.67 MA_3_Bi_2_I_9_: 2.1	PCE = 0.152%.	–

**Figure 9:**
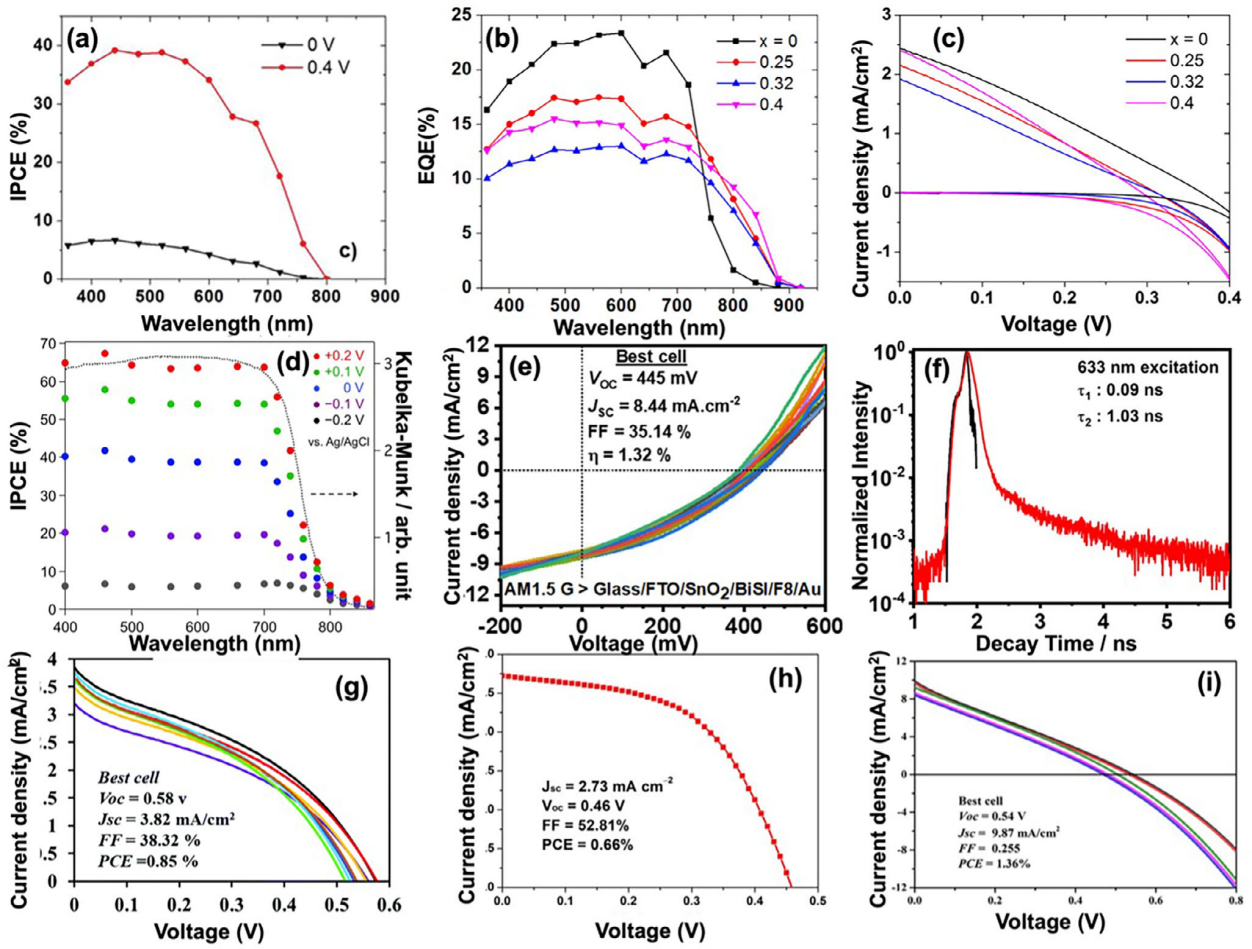
a) IPCE spectra of a film deposited at 275 °C.^[^ ^23b^ ^]^ b) Short‐circuit IPCE values measured for films with various Se/(S + Se) levels measured in a two‐electrode cell containing I^−^/I_2_ in acetonitrile.^[^ ^23a^ ^]^ c) Photoelectrochemical *I*–*V* curves for films with various Se/(S + Se) levels measured in a two‐electrode cell containing I^−^/I_2_ in acetonitrile under AM1.5G illumination.^[^ ^23a^ ^]^ d) IPCE spectra of the BiSI electrode in an acetonitrile solution containing 0.1 M NaI at various applied potentials and absorption spectrum of BiSI.^[^ ^15b^ ^]^ e) J–V characteristics of 16 devices under AM 1.5G simulated illumination and key performance metrics of the best device.^[^ ^87^ ^]^ f) Time‐resolved photoluminescence of BiSI films employing 633 nm excitation, revealing two time‐constants of 0.09 and 1.03 ns.^[^ ^87^ ^]^ g) I–V curves of 8 Bi_13_S_18_I_2_‐based solar cells.^[^ ^23c^ ^]^ h) current density–voltage (J–V) curves of BiSI.^[^ ^85^ ^]^ i) J–V curves of the five solar cell devices.^[^ ^88^ ^]^ Reproduced with permission.^[^ ^23b^ ^]^ Copyright 2012, American Chemical Society. b,c) Reproduced with permission.^[^ ^23a^ ^]^ Copyright 2012, American Chemical Society. Reproduced with permission.^[^ ^15b^ ^]^ Copyright 2016, Nature. e,f) Reproduced with permission.^[^ ^87^ ^]^ Copyright 2019, American Chemical Society. Reproduced with permission.^[^ ^23c^ ^]^ Copyright 2020, Royal Society of Chemistry. Reproduced with permission.^[^ ^85^ ^]^ Copyright 2020, American Chemical Society. Reproduced with permission.^[^ ^88^ ^]^ Copyright 2021, Wiley.

**Figure 10:**
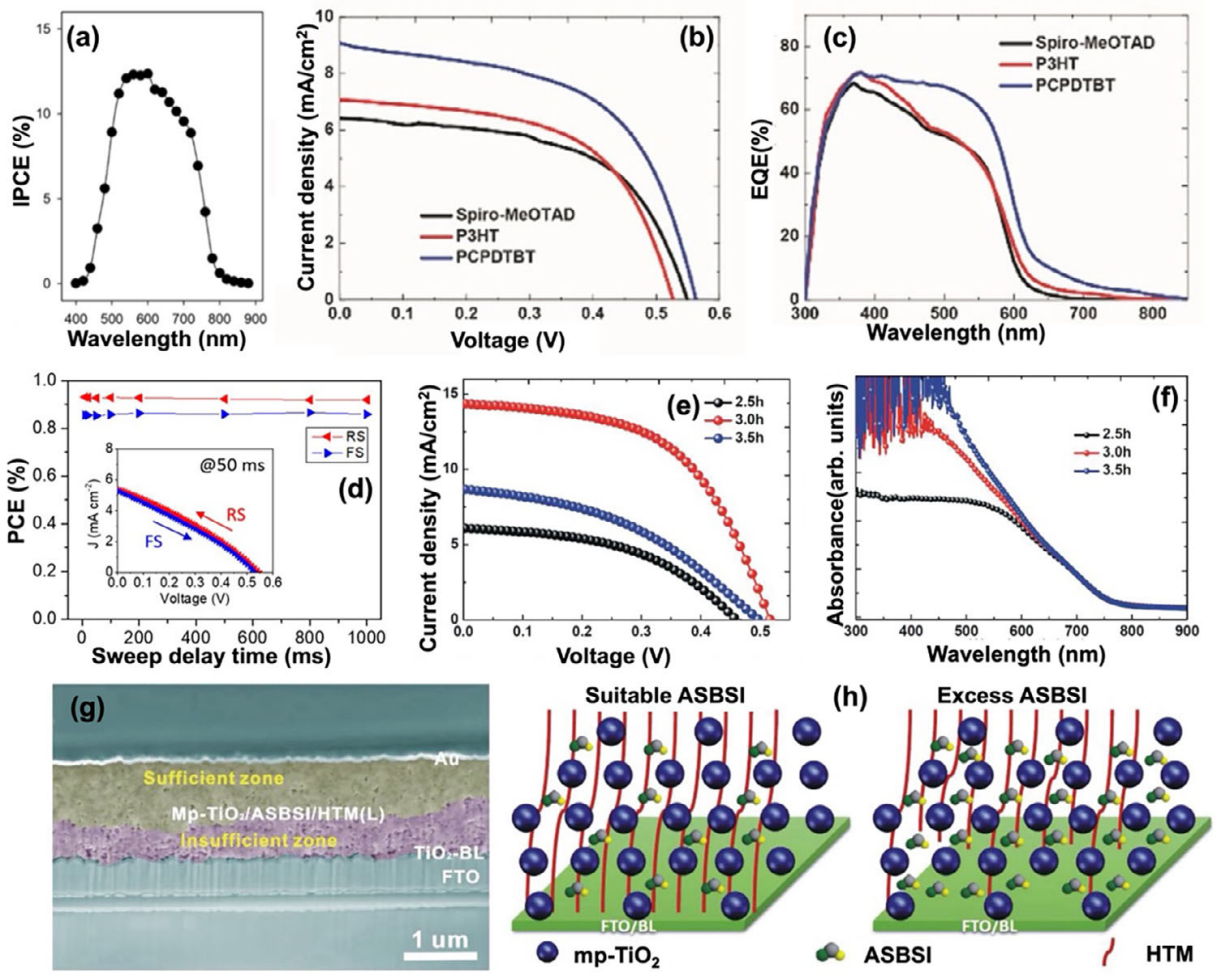
a) Incident photon to current conversion efficiency at a 0.25 V bias under monochromatic illumination.^[^ ^69^ ^]^ b) J–V curves under standard illumination conditions (100 mW cm^−2^) of air mass 1.5 global (AM 1.5G).^[^ ^23d^ ^]^ c) external quantum efficiency (EQE) spectrum of the SbSI devices with various HTMs.^[^ ^23d^ ^]^ d) photovoltaics efficiency of SbSI.^[^ ^83a^ ^]^ e,f) IPCE spectrum and UV–vis absorption spectrum of the ASBSI devices fabricated via CBD of Sb_2_S_3_ for 2.5, 3.0, and 3.5 h, respectively.^[^ ^30^ ^]^ g) The cross‐sectional FESEM image of the device fabricated via CBD of Sb_2_S_3_ for 3.5 h.^[^ ^30^ ^]^ h) Schematic diagram to explain the effect of the ASBSI amount on the infiltration of HTM.^[^ ^30^ ^]^ Reproduced with permission.^[^ ^69^ ^]^ Copyright 2022, Wiley. b,c) Reproduced with permission.^[^ ^23d^ ^]^ Copyright 2018, Wiley. Reproduced with permission.^[^ ^83a^ ^]^ Copyright 2018, AIP publishing. e–h) Reproduced with permission.^[^ ^30^ ^]^ Copyright 2019, Wiley.

The first heavy pnictogen photoelectrochemical (PEC) solar cell, utilizing BiSI as the absorber, was fabricated by Mullins and co‐workers using a single‐source chemical spray pyrolysis method in 2012.^[^ ^23b^ ^]^ Although an incident photon to current efficiency (IPCE) of ≈38% was achieved at 500 nm with a short circuit current density (*J*_sc_) of 5 mA cm^−^^2^ and an onset potential of −0.25 V (**Figure** **9**a), the PEC device exhibited stability issues, likely due to the electrochemical instability of BiSI and oxidative dissolution mechanisms. To address this, the authors adjusted the doping ratio between S and Se to realize multiple ratios of BiS_1−x_Se_x_I film, which showed tunable band gaps ranging between 1.63 to 1.48 eV (Figure 9b).^[^ ^23a^ ^]^ While this modification led to a change in crystal morphology from large micrometer‐scale rods to smaller cube‐like structures, it increased the charge carrier recombination, resulting in very low J_sc_. Moreover, the Se‐doped films exhibited worse defect‐mediated shunting behavior than undoped films, greatly limiting V_oc_ and fill factor (FF) and thereby the final device performance (Figure 9c). In 2016, Abe and co‐workers synthesized BiOI particles through a soft chemical method and fabricated BiSI electrodes via electrophoretic deposition (EPD) followed by annealing in 5% H_2_S/Ar (60 mL min^−1^) atmosphere at 100–200 °C for 1 h.^[^ ^15b^ ^]^ The PEC device based on a BiSI absorber layer with an indirect band gap of 1.51 eV showed an improved IPCE of 64% at 700 nm at +0.2 V vs Ag/AgCl) compared to previous reports (Figure 9d).^[^ ^15^, ^23^ ^]^ Later, Fermín and colleagues reported a single‐step spin coating method to deposit BiSI thin films. The deposition of BiSI thin film with flake‐shaped grain was carried out using Bi(NO_3_)_3_.5H_2_O (0.5 M), thiourea (1 M), and ammonium iodide (1 M), then precursor dissolved in a 2‐methoxyethanol: acetylacetone (4:1 parts) solvent mixture followed by heating at 200 °C in an air for 5 min. The fabricated solar cell with a device configuration of glass/FTO/SnO_2_/BiSI/F8/Au/F8 showed a record PCE of 1.32%, J_sc_ of 8.44 mA cm^−2^, V_oc_ of 0.445 V and FF of 35.14% (Figure 9e).^[^ ^87^ ^]^ However, the time‐resolved photoluminescence decay (TRPL) showed ineffective charge collection and separation due to short carrier lifetimes, coupled with high series resistance and low shunt resistance, which could restrict V_oc_ and J_sc_ (Figure 9f). Although the record efficiency of 1.32% was achieved for BiSI, poor charge collection and separation efficiencies highlight the need to find suitable alternatives for charge transport layers to achieve better band alignment with the BiSI layer.^[^ ^87^ ^]^ In the same year, it was demonstrated that a facile two‐step spin‐coating method, by depositing and annealing Bi_2_O_3_‐thiourea (Step 1) and chemically react BiI_3_ (Step 2) to form pure Bi_2_S_3_ and pure BiSI separately on TiO_2_‐BL/FTO. Results showed when Bi:S = 1:3, with the spin‐coating's repetition for two times and annealing at 200 °C created the best cell with 60–100 nm diameter nanorods along with 0.1 mA cm^−2^ and 0.4 mV structured FTO/TiO_2_/BiSI/P3HT/Au.^[^ ^83b^ ^]^ It has been analyzed that poor solar cell efficiency comes from 1) a higher energy level of ETL than the absorber layer leads to poor electron transfer and 2) the gap between HTL's HOMO and E_f_ is 0.6 eV, limiting the maximum V_oc_ to 0.6 V. Explained future efficiency could be improved by changing ETL and HTL to materials with better‐matched energy levels.^[^ ^83b^ ^]^ Many attempts have been made to improve BiSI solar cells, as the ease of converting between BiSI and Bi_13_S_18_I_2_ via changing the ratio between sulfide and bismuth offers promise. Inspired with this, Feng and co‐workers fabricated a Bi_13_S_18_I_2_ PEC device with a structure of FTO/TiO_2_(dense)/TiO_2_(porous)/ Bi_13_S_18_I_2_/(I_3_^−^/I^−^ redox)/Pt coated glass which showed a PCE of 0.85%, J_sc_ of 3.82 mA cm^−2^, V_oc_ of 0.58 V and FF of 38.32% (Figure 9g).^[^ ^23c^ ^]^ However, the reported low efficiency of 1D rod‐structured thin films with suboptimal thickness of absorber, ETL and HTL could be attributed to disorder, which increases the chance of carrier recombination in the absorber layer. Later, pioneering work by Lei and co‐workers reported the 1‐D nanostructure arrays of BiSI using a two‐step solvothermal method.^[^ ^85^ ^]^ By formation of Bi_2_S_3_ and BiSI nanorod arrays inside the autoclave sequentially. The solid‐state solar cell with a device structure of ITO/p‐CuSCN/n‐BiSI/W showed a PCE of 0.66% (Figure 9h), which was 50 times higher than the PEC device fabricated with same absorber material attributed to the perpendicular alignment of BiSI nano‐rods with the ITO substrate.^[^ ^23a^ ^]^ Inspired by Lei's work, Feng's group synthesized the first BiSCl solar cell with a structure of FTO/TiO_2_/BiSCl/(I_3_^−^/I^−^)/Pt, which showed a PCE of 1.36%, J_sc_ of ≈10 mA cm^−2^ and a relatively low V_oc_ of 0.54 V (Figure 9i).^[^ ^88^ ^]^ The high J_sc_ contributed by short nanorods and a uniform/paralleled nanorods array, which decreased the light traps and increased anti‐reflective effects, while the relatively low V_oc_ was caused by poor interface engineering between TiO_2_/(I_3_^−^/I^−^) redox and BiSCl.^[^ ^88^ ^]^ Many of attempts to prepare BiSI solar cells are based on a direct growth of an absorber layer on the conducting substrate, where adjusting the morphology of absorber material is difficult. Therefore, recent research has focused on a processable post‐synthesis method by colloidal synthesis via hot‐co‐injection of both the chalcogen and halogen precursors in a Bi‐carboxylate complex solution to prepare BiSX nanocrystals (NCs).^[^ ^69^ ^]^ The resulting BiSX (X = Cl, Br, I) NCs showed indirect bandgaps of 2.05 eV for BiSCl, 1.95 eV for BiSBr, and 1.50 eV for BiSI NCs, while direct band gap were close due to large coefficients, with the values of 2.55 eV for BiSCl NCs, 2.25 eV for BiSBr NCs, and 1.60 eV for BiSI NCs. Using hot‐co‐injection followed by double ligand exchange and annealing, a BiSI photoelectrode with metallic cluster and a reduced rugosity was fabricated to further test their potential in solar cell devices. The BiSI photoelectrode demonstrated a stable photocurrent density and more than 10% of IPCE in visible spectrum (**Figure** **10**a), showing promise as absorber layer in solar cell devices.^[^ ^69^ ^]^ BiEX is one of the most extensively studied chalcohalide PV absorbers. However, all BiEX solar cells suffer from low V_oc_ and FF, primarily due to poor thin‐film morphology. Notably, a BiSI solar cell studied by Fermin et al.^[^ ^87^ ^]^ exhibited an impressive J_sc_ of ≈8.44 mA cm^−^^2^. This can be attributed to the presence of flake‐shaped grains densely packed into highly compact structures extending over several micrometers. Despite excellent band alignment of the HTL and ETL, the V_oc_ remains relatively low, indicating that defect‐related recombination is the dominant loss mechanism. Therefore, promoting oriented crystallization and incorporating a seed layer for vertical crystal growth could be one of the key strategies for improving device performance.

**Figure 11:**
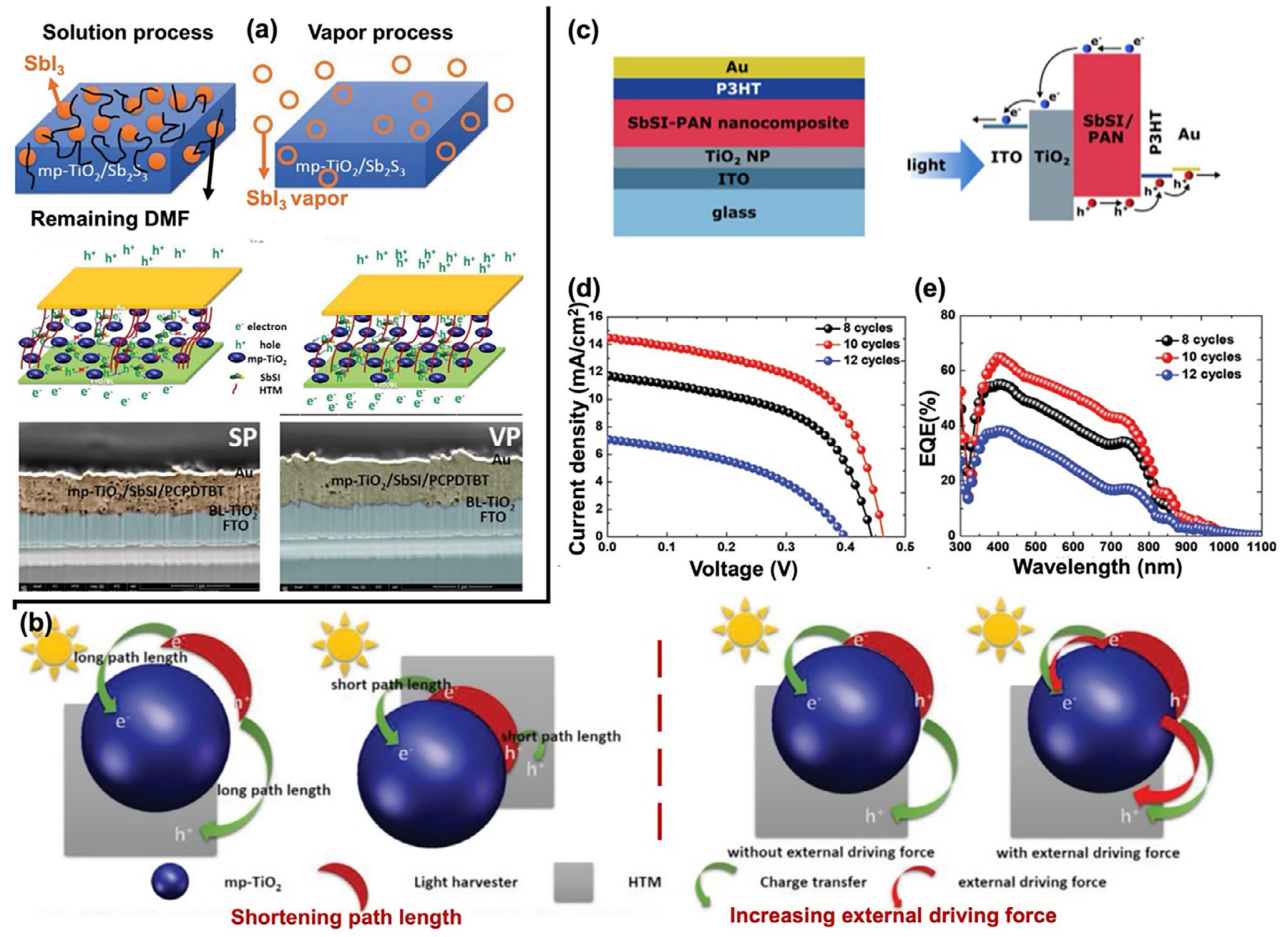
a) the schematic comparison of solution process and vapor process in formation mechanism, operating principle and cross‐sectional SEM image.^[^ ^89^ ^]^ b) two ways to enhance charge transfer.^[^ ^89^ ^]^ c) The architecture of photovoltaic device containing SbSI‐PAN nanocomposite (left) and its energy level diagram (right).^[^ ^82^ ^]^ d) J–V curves under standard illumination conditions (100 mW cm^−2^) of AM 1.5 G and e) IPCE spectra of SbSeI solar cells fabricated through 8, 10, and 12 spin‐coating cycles and thermal decomposition.^[^ ^90^ ^]^ a,b) Reproduced with permission.^[^ ^89^ ^]^ Copyright 2022, Wiley. Reproduced with permission.^[^ ^82^ ^]^ Copyright 2020, Elsevier. d,e) Reproduced with permission.^[^ ^90^ ^]^ Copyright 2021, Wiley.

Within a decade, significant progress has been made not only with BiEX but also with Sb(S,Se)I. In 2017, Seok and colleagues fabricated the first SbSI solar cell.^[^ ^23d^ ^]^ They initially synthesized Sb_2_S_3_ using a chemical bath deposition (CBD), followed by spin coating of SbI_3_ solution and further annealing at 90 °C to form SbSI. SbSI showed a direct bandgap of 2.15 eV and p‐type properties. The fabricated solar cell device with a structure of FTO/BL‐TiO_2_/mp‐TiO_2_/SbSI/PCPDTBT/Au showed a PCE of 3.05%, J_sc_ of 9.11 mA cm^−2^, V_oc_ of 0.58 V and FF of 31% (Figure 10b). Interestingly, PCPDTBT not only effectively transported holes but also absorbed long wavelength light, contributing to J_sc_, which reached ≈10 mA cm^−2^ (Figure 10b,c). Additionally, the cell demonstrated excellent stability, retaining 93% of initial PCE after 15 days without encapsulation. The following year, Choi et al.^[^ ^83a^ ^]^ employed a facile two‐step solution process to fabricate SbSI with controlled crystal growth at a low temperature of 200 °C. A planar insulating‐polymer‐free solar cell device with a structure of FTO/TiO_2_/SbSI/P3HT/Au showed PCE of 0.93%, J_sc_ of 5.45 mA cm^−2^, V_oc_ of 0.548 V and FF of 31%. Adjusting the ratio of SbCl_3_:thiourea (TU), annealing time and temperature, and SbSI nucleation pressure controlled SbSI planar morphology. The best SbSI absorbers were formed by SbCl_3_:TU = 1:3, annealed at 150 °C for 5 min, used N‐methyl‐2‐pyrrolidone (NMP) as SbI_3_ solvent, and low pressure for nucleation and crystallize. This led to the formation of a compact and uniform microstructure, mitigating leakage currents caused by voids present in the SbSI layer (Figure 10d).^[^ ^70^ ^]^ In the same year, a mixed sonication–heating method was reported for the first time to fabricate SbSI solar cells.^[^ ^81^ ^]^ Sb, S, and I powder (ratio 1:1:1) were added to ethylene glycol, followed by heating at 110 °C and stirring at 400 rpm overnight. Raman studies confirmed the formation of single crystalline NCs rods, exhibiting specific frequencies and symmetries. A solar cell device was fabricated with a structure of FTO/TiO_2_‐BL/mp‐TiO_2_/SbSI/ZrO_2_/ mesoporous carbon structure (**Figure** **11**a), yielding a PCE of 0.035%, J_sc_ of 0.4 mA cm^−2^, V_oc_ of 0.29 V, FF of 31%, with 50% retention of its original performance after 3 months of storage in laboratory condition. Compared with SbSI (1.83 eV),^[^ ^30^ ^]^ BiSI has a narrower bandgap (1.51 eV),^[^ ^37^ ^]^ potentially resulting in higher PCE by combining the high stability of Sb_2_S_3_ with the high efficiency offered by the ns^2^ configuration in BiI_3_. Taking this into account, Seok and co‐workers reported Bi‐doped SbSI thin film using a two‐step solution process. CBD was used to deposit Sb_2_S_3_, followed by a spin coating of BiI_3_ solution and heat‐treatment to form Sb_0.67_Bi_0.33_SI (ASBSI).^[^ ^30^ ^]^ Interestingly, this device showed a record efficiency of 4.07%, with a J_sc_ of 14.54 mA cm^−2^, a V_oc_ of 0.53 V and FF of 52.8%. It was revealed that reaction time during CBD process was crucial, affecting light‐harvesting efficiency (LHE) and charge transfer yield (CTY). A shorter deposition time decreased LHE, while a longer time reduced CTY due to mp‐TiO_2_ pores being filled by ASBSI, hindering proper penetration of HTM material, as confirmed by I–V curve, UV–vis spectrum, TRPL and scanning electron microscopy (SEM) analyses (Figure 10e–h). Subsequently, the same group employed the CBD method to deposit Sb_2_S_3_, followed by simple annealing and exposure to SbI_3_ vapor.^[^ ^89^ ^]^ The device with a uniform absorber layer exhibited an enhanced PCE of 3.62%. Compared to the solution process demonstrated in their previous work the vapor process developed in this work reduced charge recombination and extended carrier lifetimes due to higher absorber crystallinity and fewer pores within the absorber.^[^ ^23^, ^30^, ^89^ ^]^ This was attributed to the solvent remaining in the absorber layer, providing adhesive and gravitational forces that hindered SbSI growth. In contrast, the vapor process allowed SbI_3_ more freedom to grow (Figure 11a). Interestingly, authors used halide treatment to enhance the charge transfer by shortening path length from the light harvester to ETL/HTL and increasing the external driving force of Sb_2_S_3_ (Figure 11b). Results showed by adding SbSI layer, lower PL and reduced dark current were found in FTO/TiO_2_‐BL/mp‐TiO_2_/Sb_2_S_3_‐SbSI/PCPDTBT/Au mixed‐chalcohalide solar cell, resulting in a higher PCE of 6.08%.^[^ ^89^ ^]^ A solar cell with a structure of ITO/TiO_2_(nanoparticles)/SbSI‐polyacrylonitrile(PAN)/P3HT/Au exhibited an average J_sc_ of 1.84 µA cm^−2^ with V_oc_ of 69 mV, as shown in Figure 11c.^[^ ^82^ ^]^ Future attempt could focus on making the nanowires perpendicular to electrodes to increase the V_oc_ and J_sc_.

In contrast to SbSI, SbSeI exhibits the same A(V)B(VI)C(VII) structure with a lower bandgap (1.67 eV) and similar ferroelectric properties, showing the potential to be a promising solar absorber.^[^ ^91^ ^]^ In 2021, Seok and co‐workers developed a first SbSeI solar cell. They first thermally deposited Sb_2_Se_3_, followed by spin‐coating the Se‐SSP and multiple spin‐coatings of SbI_3_ solution to fabricate the SbSeI absorber layer. The spin‐coating cycle significantly affected the EQE, with insufficient spin‐coating cycle leading to lower absorption but efficient charge transfer. Conversely, excessive spin‐coating cycles led to an excess of SbSeI, resulting in sufficient absorption and inefficient charge transfer due to deep level traps that comes from lack of HTM penetration. The solar cell based on this SbSeI absorber exhibited the highest PCE among Sb‐based chalcohalide solar cells, with a PCE of 4.1%, J_sc_ of 14.8mA cm^−2^, V_oc_ of 473.0 mV, and FF of 58.7% (Figure 11d,e). Moreover, this material demonstrated great stability in humidity, thermal and UV environments.

**Figure 12:**
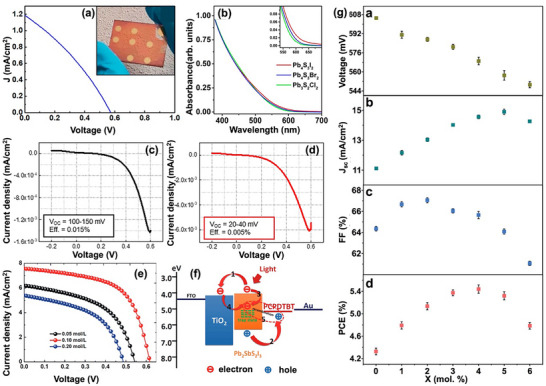
a) Current–voltage curve under AM1.5G illumination for a solar cell Inset: Photo of a representative sample.^[^ ^23i^ ^]^ b) Optical absorption spectra of the three samples.^[^ ^23i^ ^]^ c) Absorption spectra of Ag_3_SBr and Ag3SI films by PDS d) Bandgap as a function of x (Br amount).^[^ ^23j^ ^]^ e) J–V curves under standard test condition (STC).^[^ ^31a^ ^]^ f) Schematic diagrams of energy levels for the device to describe the role of trap states.^[^ ^31a^ ^]^ g) (a) open‐circuit voltage, (b) short‐circuits current density, (c) fill factor, and (d) power conversion efficiency of the Au|PTAA|Ag_3_BiI_6−2x_S_x_ | m‐TiO_2_|c‐TiO_2_|FTO solar cells.^[^ ^31b^ ^]^ a,b) Reproduced with permission.^[^ ^23i^ ^]^ Copyright 2020, American Chemical Society. c,d) Reproduced with permission.^[^ ^23j^ ^]^ Copyright 2024, Royal Society of Chemistry. e,f) Reproduced with permission.^[^ ^31a^ ^]^ Copyright 2018, American Chemical Society. Reproduced with permission.^[^ ^31b^ ^]^ Copy 2019, Wiley.

Some transition and post‐transition chalcohalide solar cells have attracted attention due to their anti‐perovskite structures and ideal bandgaps for maximum light absorption.^[^ ^23^, ^90^ ^]^ Toso and co‐workers reported the colloidal synthesis of Pb_4_S_3_Br_2_, Pb_3_S_2_Cl_2_, and Pb_4_S_3_I_2_ NCs.^[^ ^23i^ ^]^ Notably this is the first report on the synthesis of Pb_4_S_3_Br_2_ NCs resembling Pb_4_S_3_I_2_ in their Pnma orthorhombic crystal structure and featuring kinetically trapped metastable phases under normal conditions.^[^ ^23^, ^24^, ^41^ ^]^ To optimize the efficiency of Pb_4_S_3_Br_2_ solar cells, the author used ammonium (NH_4_SCN) and 1‐ethyl‐3‐methylimidazolium iodide (EMII) to convert the long chain ligands into short ligands. The improved conductivity of the Pb_4_S_3_Br_2_ thin film, with a device structure of ITO/AZO/Pb_4_S_3_Br_2_/MoO_x_/Au, displayed a PCE of 0.21% ± 0.02%, J_sc_ of 1.2 mA cm^−2^, and V_oc_ of 0.57 V (**Figure** **12**a). The low PCE could be explained by a large indirect bandgap of 1.91 eV, the absence of photoluminescence in the range of 500–1700 nm (Figure 12a,b). Ag‐based metal chalcohalides have gained attention due to their abundance and non‐toxicity. Ivan Caño and his teammates recently employed a new, facile, and low‐temperature method to synthesize anti‐perovskite structured Ag_3_SI and Ag_3_SBr solar cells with narrow bandgaps of 0.88 and 0.97 eV, achieving PCEs of 0.005% and 0.015%, respectively (Figure 12c,d). DFT calculations indicate that the presence of disorder and defects in Ag_3_SX materials change the density of states, introducing deep‐level trap states that can significantly enhance non‐radiative recombination losses.^[^ ^23j^ ^]^ Additionally, the highly porous structure of Ag_3_SX can impede charge extraction and transport by hindering the formation of percolation pathways, leading to increased resistive losses and reduced overall device performance.

One of the most effective approaches to surpassing the Shockley–Queisser limit is through tandem solar cells. Si‐based solar cells are among the best choices for the bottom cells due to their low cost and high efficiency. Some Sb‐ and Bi‐based solar cells exhibit a bandgap ≈1.68 eV, making them well‐suited as top‐cell absorbers. Examples include BiSI (1.63 eV), SbSI (1.76 eV), and SbSeI (1.78 eV), which show promises for fabricating four‐terminal tandem devices. However, further investigations into the optical transmittance and interfacial properties of chalcohalide materials are needed to identify suitable candidates for tandem integration.

**Figure 13:**
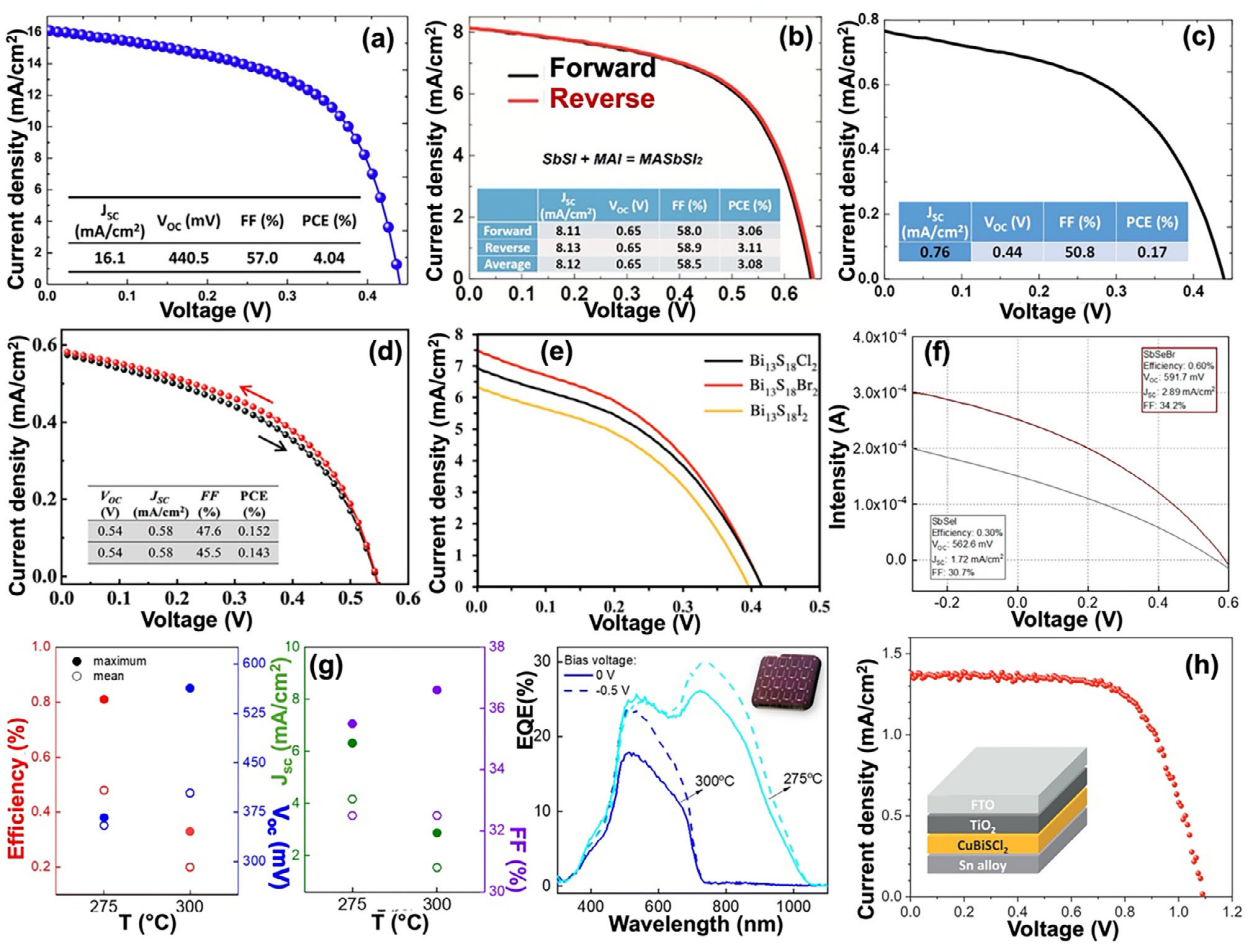
a) Current density–voltage curve of the champion Sn_2_SbS_2_I_3_ solar cell.^[^ ^31b^ ^]^ b) J–V curves in forward‐ and reverse‐scan modes of the champion MASbSI_2_.^[^ ^49^ ^]^ c) The J–V curves without MASbSI_2_.^[^ ^49^ ^]^ d) Forward and backward scanning of the reaction 30 min of MA_3_Bi_2_I_9−2x_S_x_.^[^ ^48a^ ^]^ e) J–V curve of Bi_13_S_18_X_2_ with optimal Bi_13_S_18_X_2_ thickness.^[^ ^38^ ^]^ f) intensity–voltage curves of SbSeBr and SbSeI prototype solar cells.^[^ ^40^ ^]^ g) Optoelectronic parameters of MoSbSeI/CdS/i‐ZnO/ITO solar cells under different annealing conditions: PCE and V_oc_ (left), J_sc_ and FF (middle), EQE (right) (with applied 0 V and −0.5 V).^[^ ^40^ ^]^ h) J–V curve measured on a CuBiSCl_2_ based solar cell. The inset shows the schematic structure of the device.^[^ ^23h^ ^]^ Reproduced with permission.^[^ ^31b^ ^]^ Copyright 2019, Cell. b,c) Reproduced with permission.^[^ ^49^ ^]^ Copyright 2018, American Chemical Society. Reproduced with permission.^[^ ^48a^ ^]^ Copyright 2019, Wiley. Reproduced with permission.^[^ ^38^ ^]^ Copyright 2021, American Physical Society. f,g) Reproduced with permission.^[^ ^40^ ^]^ Copyright 2023, Royal Society of Chemistry. Reproduced with permission.^[^ ^23h^ ^]^ Copyright 2019, Wiley.

Quaternary mixed chalcohalide solar cells have attracted a tremendous interest in the scientific community due to the use of metals with ns^2^ electronic configuration, compositional flexibility, and ferroelectric property.^[^ ^23^, ^31^ ^]^ In 2016, Kanatzidis and co‐workers used isothermal heating to fabricate Pb_2_BiS_2_I_3_, Sn_2_BiS_2_I_3_, and Sn_2_BiSI_5_ semiconductors with suitable bandgap,^[^ ^43^ ^]^ demonstrating the potential of these quaternary compounds as absorber in solar cells. The first quaternary chalcohalide solar cells, Pb_2_SbS_2_I_3_, were demonstrated in 2018 by Seok and co‐workers, following their two‐step solution process employed to develop Sn_2_SbS_2_I_3_ solar cells with a slight modification.^[^ ^23^, ^31^ ^]^ The Pb_2_SbS_2_I_3_ solar cell with a device configuration of FTO/BL‐TiO_2_/mp‐TiO_2_/Pb_2_SbS_2_I_3_/PCPDTBT/Au showed a PCE of 3.12%, J_sc_ of 8.79 mA cm^−2^, V_oc_ of 0.61 V, and FF of 58.2%. By investigating the electron activation energy under illumination, the authors revealed that the PbI_2_ concentration of 0.1 mol L^−1^ has the lowest trap state and recombination rates (Figure 12e). Further analysis indicated that the presence of sub‐bandgap states and traps reduces the PCE as shown in Figure 12f. Electrons may get captured by process 3. Those in the TiO_2_ might transfer back to the trap states (process 4), and electrons held in sub‐bandgap states are likely to recombine with holes in PCPDTBT (process 5). In a different study, Simonov's group made further advancement by substituting 4% of I^−^ into S^2−^, to address issues with the valence band of Ag_a_Bi_b_I_a+3b_ and the narrow bandgap of AgBiS_2_.^[^ ^31b^ ^]^ A low‐temperature solution process was employed by mixing appropriate ratios of Ag and Bi in dimethyl sulfoxide (DMSO), dimethylformamide (DMF) and hydroiodic acid (HI), adding Bi(S_2_CAr)_3_ to modify the ratio of elements in material, after which followed spinning coating and annealing in argon atmosphere (helps evaporate unwanted solvent and hasten saturation of the solution, leading to better grain size and coverage).^[^ ^31b^ ^]^ The final device with a structure of Au|PTAA|Ag_3_BiI_5.92_S_0.04_|m‐TiO_2_|c‐TiO_2_|FTO showed a PCE of 5.56%, J_sc_ of 14.7 mA cm^−2^, FF of 65.9% and a relative lower V_oc_ of 0.573 V, which could originate from the growth in size with the extent of sulfide modification can presumably act as recombination sites (Figure 12g). Inspired by this report, Nie et al.^[^ ^23e^ ^]^ in 2020, reported the first Sn_2_SbS_2_I_3_ solar cell with a device structure of glass/BL‐TiO_2_/mp‐TiO_2_/Sn_2_SbS_2_I_3_/PCPDTBT/ PEDOT:PSS/Au, yielding a PCE of 4.04%, J_sc_ of 16.1 mA cm^−2^, V_oc_ of 0.44 V, and FF of 57%. The unencapsulated device exhibited good stability at a high temperature and humidity, and under illumination (**Figure** **13**a).

Organic–inorganic lead halide perovskite solar cells (OIHPSCs) have gained huge improvement from 3.8% in 2009 to more than 25.5% up to date, inspiring research into analogous organic–inorganic lead free chalcohalides due to their environmental friendliness, high stability, optimal band gaps, high absorption efficiency and low effective mass of OIHPSCs.^[^ ^92^ ^]^ In 2018, Seok's group reported a cubic perovskite structure MASbSI_2_ with ideal tolerance factor (0.99) and E_g_ of 2.03 eV. The device based on MASbSI_2_ absorber layer showed PCE of 3.08%, J_sc_ of 8.12 mA cm^−2^, V_oc_ of 0.65 V, and FF of 58.5% when utilizing mp‐TiO_2_ as ETL, PEDOT: PSS and PCPDTBT as HTL (Figure 13b).^[^ ^49^ ^]^ The device retained 90% of initial PCE after stored in 60% ambient conditions with unencapsulated cells. Interestingly, the low band gap HTM demonstrated a separate efficiency of 0.17% for absorbing light, as evidenced by the 600–850 nm EQE spectrum (Figure 13c). In the same year, Ma and co‐workers replaced Sb with Bi and used similar method to fabricate MABiI_2_S with a more suitable bandgap of 1.52 eV, but the solar cell device yielded a lower PCE of 0.13%.^[^ ^50^, ^65^ ^]^ This is attributed to a bad morphology or leakage from nanoparticles defect sites or unsatisfactory tolerance factor (0.853) and octahedral factor (0.495).^[^ ^50^, ^93^ ^]^ In an another study, low‐pressure vapor‐assisted solution process was used to fabricate MA_3_Bi_2_I_9−2x_S_x_ thin films with improved morphology and better control over doping (sulfide) concentration.^[^ ^48a^ ^]^ The solar cell device based on an optimized absorber layer showed a PCE of 0.15% (Figure 13d).

The solution processing of chalcohalide solar cells remains in its early stages and lacks the extensive post‐fabrication optimizations that have been successfully implemented in perovskite solar cells. One of the key challenges faced by most chalcohalide devices is the presence of high defect densities, which contribute to significant losses in both V_oc_ and J_sc_ due to increased recombination. To overcome these challenges, valuable insights can be drawn from perovskite research, particularly in the areas of band engineering and GBs management, to reduce trap states and enhance charge carrier dynamics. Moreover, optimizing the precursor‐solvent systems offers an opportunity to improve uniformity and quality of the absorber layer. Advanced post‐deposition treatments, such as defect passivation and interface modifications, could play a pivotal role in improving film quality and minimizing recombination losses.

### 5.2 Vacuum Processes

Vacuum processes, on the contrary to the solution processes, offer several advantages including high‐purity films with precisely controlled thicknesses, scalability over large areas, solvent‐free processing and facile integration with tandem solar cell, despite often being associated with higher costs.^[^ ^94^ ^]^ The fabrication of Bi_13_S_18_X_2_ (X = Cl, Br, I)‐based solar cells were reported by using a facile pulsed vapor deposition (PVD) process.^[^ ^38^ ^]^ They utilized a thermal evaporator to deposit Bi_13_S_18_X_2_ onto FTO/TiO_2_‐dense electrodes. The best PCE values for FTO/TiO_2_/Bi_13_S_18_X_2_/(I^−3^/I^−^ redox couple)/Pt were found to be 1.12%, 0.91%, 0.75% for X = Br, Cl, and I, respectively (Figure 13e). The higher efficiency of the Br‐based material resulted from relatively smooth surface with some small pores. When compared with Bi_13_S_18_I_2_ fabricated by solvothermal process, vacuum‐processed device showed higher J_sc_ due to higher density and thickness of the Bi_13_S_18_I_2_ film.^[^ ^23c^ ^]^ However, solution processed solvothermal process could produce a better nanorod crystal morphology leading to better J_sc_ and FF.^[^ ^23c^ ^]^ Future optimization efforts should focus on increasing film purity to reduce the effects of trap density.

Recently, Ivan Caño and group members used high pressure technique to address the high volatility of halogen/halide compounds during vacuum processes by increasing the sublimating point of halides.^[^ ^40^ ^]^ The results showed that structures with SLG/Mo/SbSeX/CdS/ZnO/ITO/Ag (X = Cl, Br) reached PCEs of 0.6% and 0.3%, respectively (Figure 13f,g). The low PCE should be addressed by modifying contact of ETL and absorb layer as well as modifying the inefficient band structure. It was revealed that by evaluating the formation energy of the precursor powder (CuCl, Bi_2_S_3_, BiCl_3_), Ming et al.^[^ ^23h^ ^]^ have established the stability of the resulting CuBiSCl_2_, affirming its suitability for consistent fabrication. Following synthesis via the solid‐state method at 430 °C for an extended duration to ensure stoichiometric precision, CuBiSCl_2_ showcased notable enhancements in thermal and humid stability, enduring temperatures of up to 300 °C while preserving structural integrity under 60% relative humidity for 25 days. This superior stability performance surpassed that of LHPs, demonstrating exceptional resistance to both thermal and moisture challenges. The resultant unoptimized solar cell, adopting an FTO/TiO_2_/CuBiSCl_2_/Sn alloy structure, yielded PCE of 1%, J_sc_ of 1.38 mA cm^−2^, V_oc_ of 1.09 V, and FF of 66% (Figure 13h).

Currently, the lack of studies on vacuum‐processed solar cells could be attributed to several reasons: 1) the high cost of vacuum machines and their large energy consumption compared with low‐cost solution processes, 2) the challenge of precisely controlling the stoichiometric ratio of the deposited chalcohalide films using co‐evaporation method due to the difference of the evaporation rates of each precursor, which is even worse for quaternary chalcohalides,^[^ ^94^, ^95^ ^]^ 3) the mutual influence of precursor sublimation rates during co‐evaporation process, among others.^[^ ^95^ ^]^

## 6 Long‐Term Stability

Whether it can be commercialized is an important reference factor in the study of solar cells. Although OIHPSCs solar cells exhibit high efficiency, their photodecomposition and susceptibility to oxidation under conditions of moisture and oxygen have hindered their commercialization process.^[^ ^96^ ^]^ Compared with OIHPSCs, utilizing either individual metal cations such as Cu^+^, Sn^2+^, Bi^3+^, Sb^3+^, Ag^+^ or mixed‐metal cations in conjunction with mixed anions of halogens and chalcogens.^[^ ^13^, ^23^, ^96^ ^]^ The former, offering ns^2^ electron pairs similar to those of lead, not only achieves defect tolerance, high dielectric constants, and low effective masses akin to perovskite materials but also resolves the toxicity issues associated with Pb^2+^. The latter, through mixed‐anion configurations, contributes to higher chemical stability.^[^ ^13^, ^14^ ^]^ Due to stronger covalent bonds in chalcohalides compared to pure halides, and increased strength of metal–chalcogen bonding, these materials exhibit increased resistance to environmental factors such as moisture, oxygen, and light exposure.^[^ ^24c^ ^]^ For instance, Ming et al.^[^ ^23h^ ^]^ fabricated the unoptimized CuBiSCl_2_ solar cell showed good stability under 300 °C and ambient condition; Nie et al.^[^ ^23e^ ^]^ developed Sn_2_SbS_2_I_3_ solar cell could reserve more than 80% of initial PCEs after 600 h of 80% relative humidity; designed SbSeI cell reserve 90% after 40 h standard test condition (STC) continuous illumination, etc.^[^ ^90^ ^]^

The stability of solar cells depends not only on the properties of the materials themselves but also on various manufacturing methods, different layers’ material, and fabrication conditions.^[^ ^13^, ^67^ ^]^ Recently, Javier and colleagues used Bi(SCN)_3_, KSn(SO_4_)SCN or Sn(SCN)_2_ as different kinds of thiocyanate as sulfur‐source to fabricate the quaternary tin chalcohalide with different stoichiometric.^[^ ^16d^ ^]^ Results showed temperature, reaction time, and reaction precursors quantities are crucial parameter in fabrication. Suitable parameters yield pure, no second phase (binary, ternary phases) products. Further stability test (vigorously stirred each chalcohalide in water for 48 h) revealed tin‐based quaternary material are stable. They used X‐ray photoelectron spectroscopy (XPS) to test Sn_2_SbS_2_I_3_, only small shift in the Sn 3d_5/2_ from 486.4 to 486.6 eV.^[^ ^16d^ ^]^

## 7 Summary and Perspective

Chalcohalide materials represent an emerging class of semiconductors, promising to combine the most favorable application‐relevant properties such as excellent optoelectronic and defect‐tolerant properties of halide perovskites with the outstanding stability of chalcogenides. While still in the early stages of their development, promising PCEs in Sb‐based chalcohalides solar cells have spurred significant interest within the PV community. This review supports this growing interest by elucidating the fundamental properties essential for PV applications, such as structure–optoelectronic property relationships, dielectric and defect‐tolerance of this impressive family of chalcohalide materials. Our aim of this review is to highlight the critical challenges in synthesis, device structures and fabrication that must be addressed for chalcohalides to achieve high efficiency PV devices.

Below, we summarize key findings and suggest future research directions to advance understanding of chalcohalide materials and ultimately realize their full potential in solar cells.

1. Since chalcohalide materials research is still in its infancy, strengthening the theoretical understanding of their fundamental properties is essential. This includes detailed studies of widely studied Bi‐ and Sb‐based ternary chalcohalides (e.g., BiEX and SbEX) as well as emerging transition mixed‐metal‐based chalcohalides (e.g., Sn_2_BiS_2_I_3_, AgBiSCl_2_ and CuBiSCl_2_). These studies should focus on comprehensive understanding of their structure, optoelectronic behavior, dielectric properties and defect tolerance to unlock their potential for PV applications.
2. Chalcohalide materials exhibit diverse structural characteristics, ranging from 1D to 2D and even 3D frameworks, depending on their compositions. For example, the widely studied chalcohalide materials family, SbSI, primarily adopts a 1D structure, resulting in thin films with needle‐like, columnar or lamellar formations that leads to surface defects and nonuniform coverage on conducting substrates, often causing device shunting. To address these issues, engineering precursors‐solvent systems or applying post‐deposition treatments are promising approaches. Another common approach is using mp‐TiO_2_ as a ETM, however mp‐TiO_2_ suffers from poor stability under UV light and has low electron mobility compared to other ETMs used for halide perovskites. Additionally, there is often a band alignment mismatch between mp‐TiO_2_ and several chalcohalide materials, resulting in low device efficiency. Therefore, systematic studies are needed to optimize the thin film formation recipes for ternary chalcohalides (e.g., SbSX and BiSX) and to develop novel processes for preparing 2D and 3D quaternary chalcohalides (e.g., Sn_2_BiS_2_I_3_, AgBiSCl_2_ and CuBiSCl_2_), which have shown excellent optoelectronic and defect‐tolerant properties.
3. Beyond optimizing thin film crystallization, developing stable and efficient charge transport and electrode materials is critical. Currently, the highest efficiency chalcohalide solar cells use a mesoscopic device structure with mp‐TiO_2_, which has several disadvantages as previously discussed. Developing HTMs and ETMs with more appropriate energy levels and designing novel device architectures, such as SnO_2_/ZnO‐chalcohalide absorbers or HTM‐free chalcohalide devices, can greatly enhance charge transfer. The most widely adopted electrode material for chalcohalide solar cells is expensive Au, or in some cases, Ag. These can be replaced by carbon electrodes, which can improve both charge extraction and encapsulation, advancing stability and cost‐effectiveness of chalcohalide solar cells.
4. Simultaneously, utilizing systematic high‐throughput computational simulations and modelling is important to correlate experimental findings and guide device design. For example, 1D SCAPS can simulate optimized device structures, materials, and layer thicknesses, providing authoritative references for experimental studies. Additionally, Silvaco technology's computer‐aided design simulation code can calculate energy bands, photogenerated rates, J–V characteristics, and spectral responses of chalcohalide solar cells with different configurations. Beyond device simulations, theoretical investigations using first‐principle calculations, such as those implemented in the Vienna Ab initio simulation package, are essential for examining the electronic and atomic structures of chalcohalide materials and their interfaces. These theoretical investigations provide critical insights into interfacial properties through DFT calculations, enabling a deeper understanding of material behavior at the atomic level. Such insights are crucial for improving the stability and performance of chalcohalide‐based devices. For example, computational studies have predicated the bandgap tunability of Sb(S, Se)(I, Br) alloys, guiding experimental efforts in bandgap engineering for tandem solar cells.^[^ ^39^ ^]^ Adaptationally, high‐throughput computational screening using the MP database identified CuBiSCl_2_ as a promising mixed‐metal chalcohalide with a direct bandgap of ≈1.37 eV and defect tolerant characteristics.^[^ ^23h^ ^]^ This theoretical predication was experimentally validated, leading to the successful synthesis of CuBiSCl_2_ with a measured band gap of 1.44 eV and strong absorption near the band edge, aligning well with calculations. Thus, these theoretical frameworks provide the basis for understanding the fundamental properties of chalcohalides and serve as reliable references to guide experimental studies and optimize device designs.
5. These chalcohalides have shown initial promise in single junction solar cells but require extensive efforts to realize their full potential. Besides improving their single junction device performance, some chalcohalide materials are suitable as top or bottom cells in tandem solar cells. As discussed earlier, the best suited absorbers for single‐junction solar cells appear to be Sn–Bi–S–I materials with various compositions and CuBiSCl_2_ materials with ideal bands gaps of 1.2–1.4 and 1.4–1.5 eV, respectively. These materials offer the added benefits of being relatively less‐toxic and composed of earth‐abundant elements. Notably, the highest reported efficiency of 4.04% to date for chalcohalide solar cells use Sn_2_BiS_2_I_3_ as the absorber. However, its narrow compositional window may hinder achieving high efficiency solar cells. On the other hand, CuBiSCl_2_, an absorber with a relatively easy‐to‐control composition, has demonstrated an initial efficiency of 1% for an unoptimized device structure, demonstrating promise as an absorber for single‐junction solar cells. Other chalcohalide materials, such as wide band gap chalcohalides (e.g., SbSI with an *E_g_* of 2.15 eV and Ag_3_BiI_6−2x_S_x_ with an *E_g_* of 1.76–1.87 eV), have been used as absorbers in single‐junction solar cells and could potentially serve as top cells on silicon for tandem solar cells. However, their current single‐junction solar cell efficiencies are insufficient for fabricating tandem devices. With improvements in their efficiencies of single‐junction devices, these materials could become promising candidates for tandem or multijunction solar cells. Francisco Palazon concluded that all‐chalcohalide tandem devices based on Pb_4_S_3_I_2_/ Ag_3_SI exhibit a high theoretical PCE of 45%.^[^ ^24c^ ^]^ However, studies on narrow‐band‐gap chalcohalides remain elusive, possibly due to their complex synthesis processes. Further efforts should focus more on developing appropriate synthesis methods to prepare high‐quality chalcohalide materials. Additionally, engineering the interface between different layers is crucial for improving their single‐junction device efficiency and advancing their progress toward tandem device integration.

In summary, research on chalcohalide materials has seen growing interest in recent years due to their promising potential as nontoxic and earth‐abundant alternatives. They have shown promise in PV devices with initial efficiencies and have further great development prospects. However, substantial progress is required in materials synthesis, thin‐film optimization, and device architecture to rival high efficiencies of chalcogenide and halide perovskite counterparts. The challenges and opportunities presented by all chalcohalide materials coexist, pointing to a bright and exciting future for this field as further advancements are made.

## Conflict of Interest

The authors declare no conflict of interest.
